# ELF5 Drives Lung Metastasis in Luminal Breast Cancer through Recruitment of Gr1+ CD11b+ Myeloid-Derived Suppressor Cells

**DOI:** 10.1371/journal.pbio.1002330

**Published:** 2015-12-30

**Authors:** David Gallego-Ortega, Anita Ledger, Daniel L. Roden, Andrew M. K. Law, Astrid Magenau, Zoya Kikhtyak, Christina Cho, Stephanie L. Allerdice, Heather J. Lee, Fatima Valdes-Mora, David Herrmann, Robert Salomon, Adelaide I. J. Young, Brian Y. Lee, C. Marcelo Sergio, Warren Kaplan, Catherine Piggin, James R. W. Conway, Brian Rabinovich, Ewan K. A. Millar, Samantha R. Oakes, Tatyana Chtanova, Alexander Swarbrick, Matthew J. Naylor, Sandra O’Toole, Andrew R. Green, Paul Timpson, Julia M. W. Gee, Ian O. Ellis, Susan J. Clark, Christopher J. Ormandy

**Affiliations:** 1 Garvan Institute of Medical Research and The Kinghorn Cancer Centre, Darlinghurst, New South Wales, Australia; 2 St. Vincent’s Clinical School of Medicine, Faculty of Medicine, University of New South Wales, Darlinghurst, New South Wales, Australia; 3 Peter Wills Bioinformatic Center, Garvan Institute of Medical Research, Darlinghurst, New South Wales, Australia; 4 The University of Texas MD Anderson Cancer Center, Houston, Texas, United States of America; 5 Department of Anatomical Pathology SEALS, St. George Hospital, Kogarah, New South Wales, Australia; 6 School of Medicine and Health Sciences, University of Western Sydney, Campbelltown, New South Wales, Australia; 7 Sydney Medical School, University of Sydney, Camperdown, New South Wales, Australia; 8 Department of Histopathology, Nottingham City Hospital and Nottingham University, Nottingham, United Kingdom; 9 Cardiff School of Pharmacy and Pharmaceutical Sciences, Cardiff University, Cardiff, United Kingdom; Friedrich Miescher Institute, SWITZERLAND

## Abstract

During pregnancy, the ETS transcription factor ELF5 establishes the milk-secreting alveolar cell lineage by driving a cell fate decision of the mammary luminal progenitor cell. In breast cancer, ELF5 is a key transcriptional determinant of tumor subtype and has been implicated in the development of insensitivity to anti-estrogen therapy. In the mouse mammary tumor virus-Polyoma Middle T (MMTV-PyMT) model of luminal breast cancer, induction of ELF5 levels increased leukocyte infiltration, angiogenesis, and blood vessel permeability in primary tumors and greatly increased the size and number of lung metastasis. Myeloid-derived suppressor cells, a group of immature neutrophils recently identified as mediators of vasculogenesis and metastasis, were recruited to the tumor in response to ELF5. Depletion of these cells using specific Ly6G antibodies prevented ELF5 from driving vasculogenesis and metastasis. Expression signatures in luminal A breast cancers indicated that increased myeloid cell invasion and inflammation were correlated with *ELF5* expression, and increased ELF5 immunohistochemical staining predicted much shorter metastasis–free and overall survival of luminal A patients, defining a group who experienced unexpectedly early disease progression. Thus, in the MMTV-PyMT mouse mammary model, increased ELF5 levels drive metastasis by co-opting the innate immune system. As ELF5 has been previously implicated in the development of antiestrogen resistance, this finding implicates ELF5 as a defining factor in the acquisition of the key aspects of the lethal phenotype in luminal A breast cancer.

## Abstract 2

Up-regulation of the transcription factor ELF5 in tumors helps to create a micro-environment that recruits the innate immune system and increases vascular permeability, leading to increased metastasis in luminal breast cancer. Together with its role in anti-estrogen resistance, this suggests that ELF5 is a major driver of a lethal phenotype.

## Author Summary

The transcription factor Elf5 defines hormone-insensitive and endocrine-therapy–resistant breast cancer. In this study, we have discovered that ELF5 drives the spread of tumor cells to the lungs. We demonstrate that the underlying mechanism for this metastatic spread is via recruitment of the innate immune system. Interestingly, this effect is able to overcome the other tumor-suppressive effects of ELF5 on cancer cells, such as reduced proliferation, motility, and invasion. This important finding challenges the more conventional view that the most potent determinant of metastatic activity lies within the cancer cell. We clearly demonstrate that the innate immune system strongly influences the metastatic activity of cancer cells despite their cell-intrinsic spread potential. Our previous work demonstrated that in luminal breast cancer, ELF5 is a key determinant of antiestrogen therapy resistance. Here, we show that the metastatic mechanism driven by ELF5 is most important in luminal breast cancer patients, in whom higher ELF5 expression is associated with low presence of cytotoxic T lymphocytes, an immune cell population responsible for tumor rejection. Thus, we now see that ELF5 may be behind the two most important processes that cause luminal breast cancers to progress towards the lethal phenotype; resistance to antiestrogen therapy and the development of metastatic activity. This understanding could pave the way for new therapeutic strategies to be devised and new predictive tests to be developed.

## Introduction

Breast cancer is a heterogeneous disease in which subtypes predicting differential clinical outcome are recognized based on shared patterns of gene expression and mutation, indicating a shared path to cancer [1]. The most striking subtype distinction in breast cancer is provided by expression of ESR1, the estrogen receptor (ER). This divides breast cancer into two very different diseases, recognizable by more than their response to hormones and antiestrogen therapies. For example, the risk of recurrence remains constant for more than 20 y for ER+ disease, but drops dramatically after 5 y for ER- disease [2,3]. ER+ cancers are also more insensitive to chemotherapy than those that are ER- [4–6]. The basis for this phenotypic dichotomy probably includes the characteristics of the cancer’s cell of origin, which for the basal ER- and luminal ER+ breast cancer subtypes are thought to be the members of the mammary progenitor cell pool [7].

A key transcriptional determinant of cell fate decisions made by the progenitor cells is the ETS transcription factor ELF5 [8], which is first expressed as mammary stem cells differentiate to become progenitor cells, coincident with promoter demethylation [9]. In progenitor cells ELF5 levels fall under hormonal control. The systemic hormones of pregnancy prompt local mammary paracrine signals involving RANKL [10–12] to induce ELF5 [13,14], and force a progenitor cell fate decision that establishes the ER- secretory cell lineage responsible for milk production. An alternative progenitor cells fate, that of an ER+ hormone sensing cell, may result if ELF5 levels remain in check due to the dominance of the estrogen-driven phenotype [15].

In luminal breast cancer cells, a mutual negative-regulatory loop between ER and ELF5 occurs, which is dominated by ER and so keeps ELF5 levels low [16]. Conversely, ER- basal breast cancers are characterized by high ELF5 levels, while the stem-cell–like claudin-low subgroup does not express *ELF5* [16]. Knockdown of ELF5 levels in luminal breast cancer cells has a small effect on proliferation, but a much greater effect is seen in ER- basal cell lines [16]. Importantly, ELF5 levels rise when MCF7 luminal breast cancer cells acquire antiestrogen resistance, and resistant cells become dependent on ELF5 for their proliferation [16]. Thus, increased ELF5 levels provide an escape pathway from inhibition of proliferation by antiestrogen therapy, facilitating disease progression. Whether ELF5 is involved in other key aspects of disease progression, such as metastasis, is unknown.

Like primary tumor formation, the acquisition of the metastatic phenotype involves events that alter both intrinsic cell behavior and the extrinsic responses of the host environment. An example of an intrinsic event is the gain of phenotypic plasticity, which regulates the acquisition of invasive and motile characteristics to cancer cells [17]. ELF5 influences phenotypic plasticity by driving the expression of epithelial characteristics, as shown by the fact that knockout of Elf5 in mice, or knockdown of breast cancer cells, caused the loss of epithelial patterns of gene expression, while forced Elf5 expression caused their gain [16,18].

An example of an extrinsic event is the interaction of the tumor with the host immune system. For example, in the mouse mammary tumor virus–Polyoma Middle T (MMTV-PyMT) model of breast cancer, knockout of CSF-1 depleted macrophages and delayed the development of lung metastases, while over expression caused the migration of macrophages into the tumor and accelerated metastasis [19,20]. Another important innate immune cell subset active in metastasis of mammary and breast cancer are myeloid-derived suppressor cells (MDSC) [21]. Their circulating numbers are increased by the presence of a tumor [22,23]. They invade primary tumors, where they promote angiogenesis, via Matrix Metaloproteinases (MMP) secretion and Vascular Endothelial Growth Factor (VEGF) production [24]. These cells inhibit and kill natural killer cells [25] and T-cytotoxic lymphocytes [26], while promoting the proliferation of the T-regulatory cell population and inhibiting dendritic cell maturation; all mechanisms that allow tumors to evade immune control [27]. In some contexts MDSC can also promote type II macrophage development and macrophage-assisted metastasis. In the MMTV-PyMT model of mammary metastasis, increased TGF beta signaling caused their recruitment to primary tumors. Depletion of their numbers reduced the number of lung metastases while tumor cell co-inoculation with MDSC increased the number of lung metastases [28,29].

We have used our inducible mouse model of mammary-specific ELF5 expression, in the context of luminal mammary tumors induced by PyMT expression, to investigate the roles played by ELF5 during mammary carcinogenesis and progression to metastatic disease.

## Results

### Elf5-Inducible Model of Mammary Carcinogenesis

To investigate the effects of Elf5 expression in breast cancer progression, we crossed our mammary epithelial specific ELF5-inducible transgenic mouse [8] with the MMTV-PyMT mouse model of luminal mammary cancer [30–32]. Triple-transgenic animals were created carrying one copy of each of the alleles (S1A Fig) on an inbred FVB/N genetic background. Time course experiments showed that after 7 d of Doxycycline (DOX) in the feed the ELF5 protein was detectable by western blot in established mammary tumors and that expression was maintained for at least 8 wk (S1B Fig).

### Elf5 Reduces Tumor Cell Proliferation and Induces Epithelial Properties

**Fig 1:**
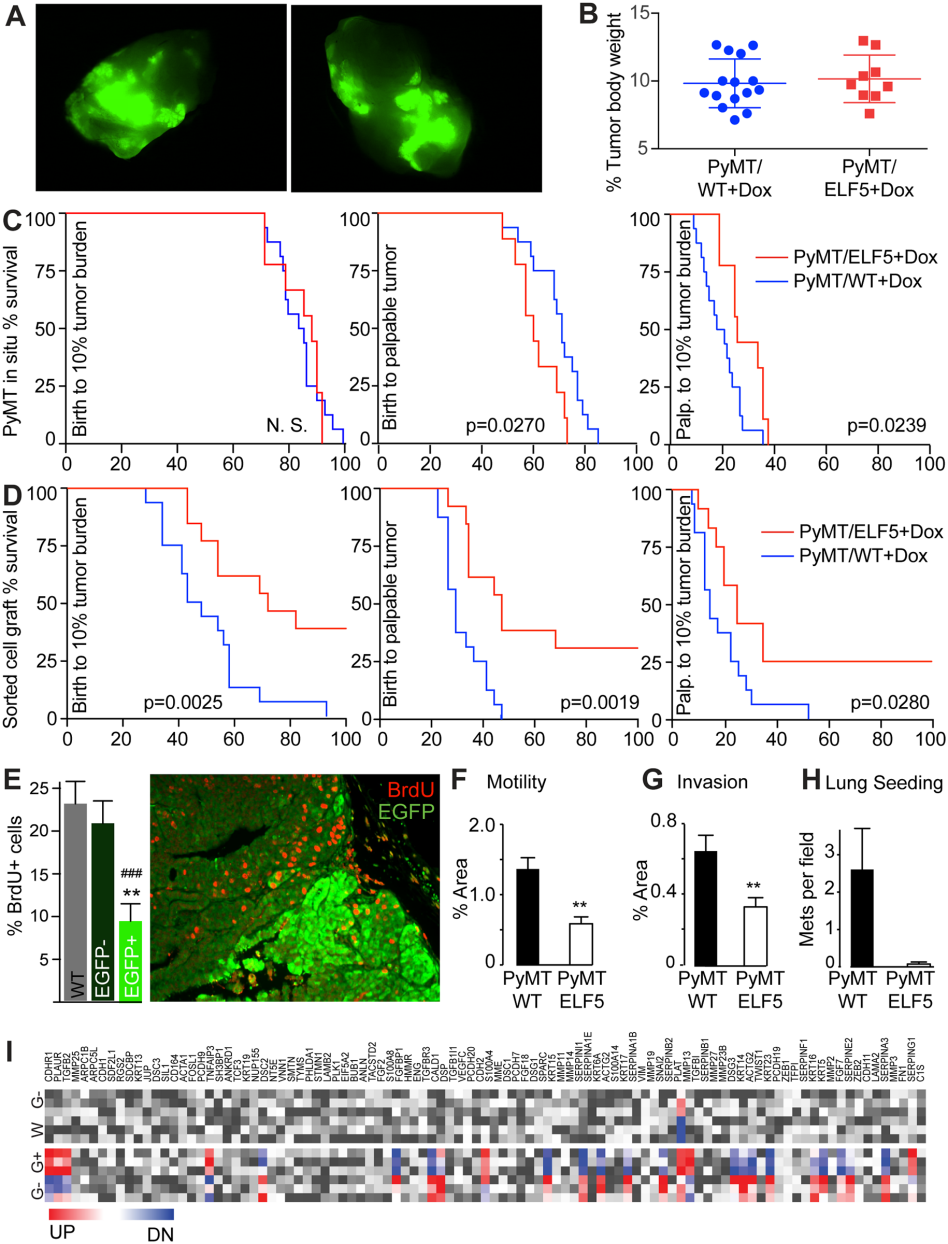
Effects of forced Elf5 expression on tumor growth and cell autonomous pro-tumorigenic traits. **Panel A**, PyMT mammary tumors showing heterogeneous expression of ELF5 visualized by EGFP expression. **Panel B**, percent primary tumor burden for each experimental group showing equivalence. **Panel C**, survival analysis of time to ethical endpoint (10% tumor burden), time to tumor detection, and time from detection to the ethical endpoint, in animals of the indicated genotypes carrying tumors that developed in situ. **Panel D**, survival analysis, as above, in animals carrying tumors that developed from an intraductal transplant of EGFP+ tumor cells derived from animals treated long term with DOX. Log-rank *p*-values are shown for +/- DOX comparison. N.S.; not significant. **Panel E**, representative image of cell proliferation measured by BrdU incorporation (red cells) in EGFP high (bright green) compared to EGFP low/no areas (dark green) of primary tumors, quantified by counting cells in random fields (bar chart). **Panels F and G**, Boyden chamber assays of motility and invasion through matrigel of EGFP high cells (PyMT ELF5) compared to wild-type PyMT cells separated by flow sorting. **Panel H**, lung colonies per field that developed from EGFP high or wild-type cells injected through the tail vein. **Panel I**, changes in gene expression of a set of genes involved in epithelial and mesenchymal characteristics. Significant increase in expression in red (UP), decrease in blue (DN), and nonsignificant changes in grey. Labels are EGFP- (G-), EGFP+ (G+) and WT (W). Raw data for panels E, F, G, and H can be found in S1 Data.

Induction of ELF5 was measured in whole tumors by imaging EGFP fluorescence. A heterogeneous pattern of expression was observed (Fig 1A), which may have resulted from a chimeric expression pattern of the *rtTA* transgene, a feature of older MTB mice [33]. We used Kaplan-Meier survival plots to analyze primary tumor growth. Only mice that showed a tumor burden of ~10% (7%–13%) of body weight at autopsy were included in the analysis (Fig 1B). Overall survival at ~10% tumor burden showed no significant difference (Fig 1C LHS), however, forced expression of *Elf5* produced tumors that were detected earlier (Fig 1C middle), but which took longer to then reach the ethical endpoint (Fig 1C right-hand side [RHS]). To overcome the effects of heterogeneous ELF5 induction (Fig 1A), we performed intraductal allografts of Fluorescence-Activated Cell Sorting (FACS)-sorted (Lin- and CD24+) tumor cells that were either EGFP (ELF5) positive or negative. Purified cells were injected into the mammary ducts of FVB/N host animals pretreated with DOX and maintained on DOX. EGFP+ transplants resulted in longer overall survival, longer time to tumor detection and longer time to the ethical endpoint, than transplants originated from EGFP- cells (Fig 1D). To demonstrate that EGFP/ELF5 was not only expressed in a particular subset within the mammary epithelium, we performed a similar experiment including allografts made from cells that were sorted (Lin- and CD24+) from excised tumors not carrying the *ELF5* transgene (PyMT/wild type [WT]) or cells that were purified from tumors (PyMT/ELF5) made fluorescent by a short 7 d pulse of DOX administration, to allow flow capture of EGFP+ cells as before, but then injected into the mammary ducts of hosts either pretreated and maintained on DOX, or not ever treated with DOX (S2A Fig). As before, EGFP+ allografts maintained on DOX produced slower growing tumors. The two control groups (WT and EGFP+ with no DOX after transplant) produced tumors that expanded at indistinguishable rates.

The effect of ELF5 on a variety of cell-autonomous endpoints was examined. Cell proliferation was analyzed using a BrdU pulse to label cells in S-phase and EGFP IF to detect Elf5-expressing areas. We observed that much higher rates of cell proliferation occurred in the areas of the tumor which expressed low levels of ELF5, marked by low or no EGFP. This was observed after 2 wk of Elf5 induction (S2B Fig) and was maintained for at least 8 wk of DOX treatment (Fig 1E), indicating long-term functional activity of the Elf5 transgene. We used these flow-sorted primary cells to examine other cell-autonomous aspects of ELF5 action in tumor formation. ELF5 reduced the motility of tumor cells through a permeable membrane in a Boyden chamber, using serum as the chemo-attractant (Fig 1F), and also reduced the ability of these cells to invade through a layer of matrigel using the same apparatus (Fig 1G). Injection of primary cells into the tail vein of wild-type hosts produced engraftment of WT tumor cells in the lungs, but rarely when the cells expressed ELF5 (Fig 1H). We compared these cell populations using Affymetrix MoGene transcript expression arrays and examined the expression of genes indicative of epithelial and mesenchymal characteristics. Long-term induction of ELF5 produced a detectable mesenchymal to epithelial transition while EGFP- cells showed no change and resembled WT PyMT cells (Fig 1I). Together these data show that forced Elf5 expression reduced cancer cell proliferation, motility, invasion and mesenchymal characteristics, corresponding with reduced primary tumor growth in the MMTV-PyMT mouse mammary cancer model.

### Elf5 Produces Hemorrhage, Leukocyte Infiltration, and Angiogenesis in MMTV-PyMT Primary Tumors

**Fig 2:**
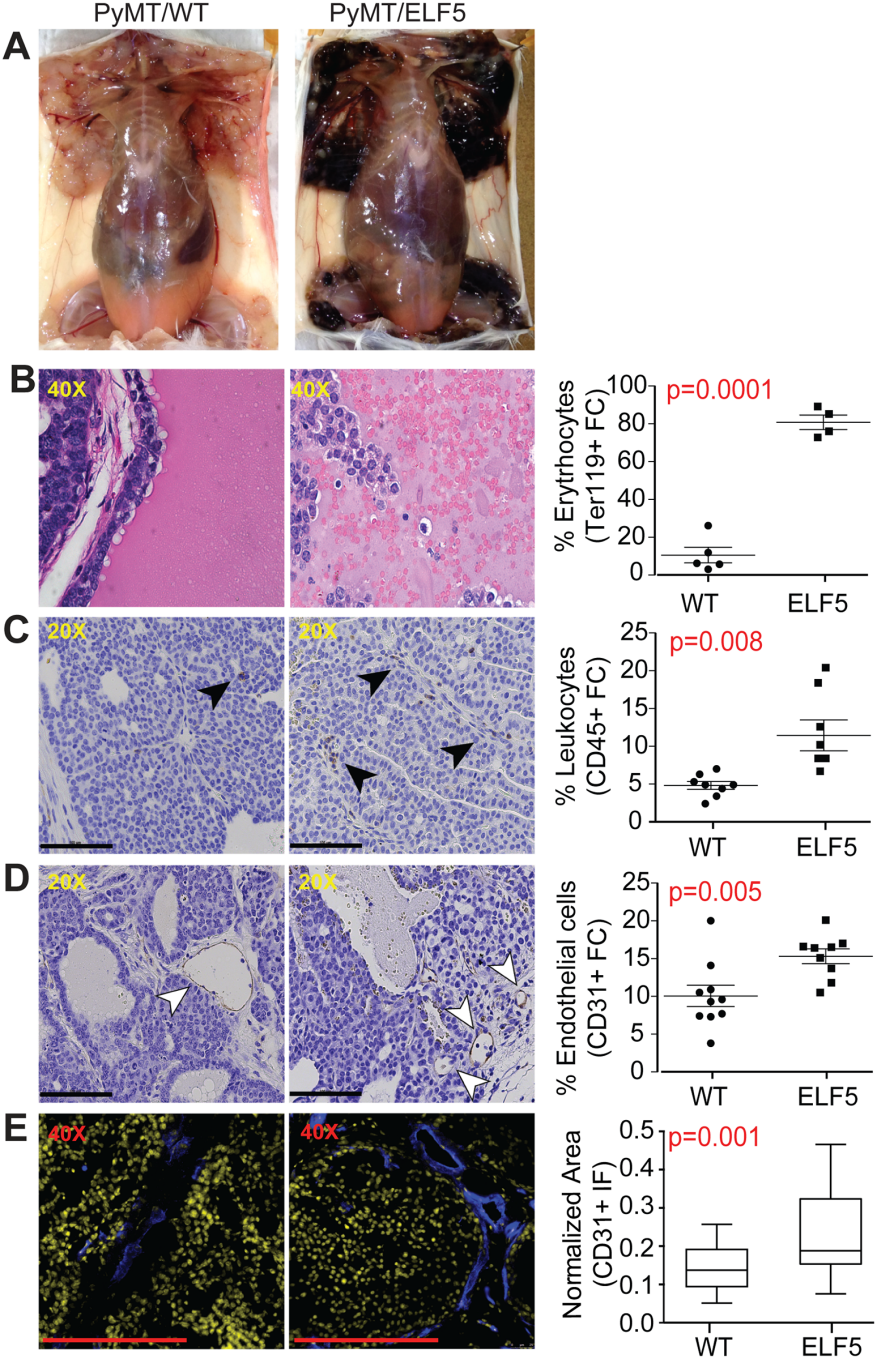
ELF5 produces hemorrhagic mammary tumors and increased tumor vasculature. **Panel A**, appearance of PyMT-driven tumors in WT mice or those experiencing long-term (8 wk) forced expression of Elf5. **Panels B–D**, increased presence of erythrocytes (H&E), leukocytes (black arrows), and endothelial cells (white arrows), respectively (immunohistochemistry [IHC]), driven by ELF5, (scale bars 100μm). Flow cytometric (FC) quantification of these effects is shown in the right-hand side (RHS) panels. **Panel E**, measurement by immunofluorescence (IF) of CD31+ endothelium area (blue) in relation to the total cell area stained by DAPI (yellow). ImageJ quantification of random fields is shown in the RHS panel.

Induction of ELF5 caused wide-spread tumor hemorrhage. This was apparent as small and discrete areas of hemorrhage after 2 wk of induction that rapidly developed to affect the entire tumor (Fig 2A). Haematoxylin and eosin (H&E) histology showed pools of erythrocytes within the affected area of the tumor and macrophages exhibiting hemosiderin (Fig 2B). Infiltrating CD45+ leukocytes were found associated as clusters or along basement membrane planes between lobular structures (Fig 2C). Quantification using flow cytometry (FC), revealed a 6-fold increase in Ter119+ tumor erythrocytes (Fig 2B RHS) and 2-fold increase in CD45+ leukocytes (Fig 2C RHS). Immunohistochemical staining for endothelium using antibodies recognizing CD31 revealed a higher vascular density with finer and more branched vessels in response to Elf5 (Fig 2D). Flow cytometry showed a 1.5-fold increase in CD31+ endothelial cell content of tumors. Quantification of endothelial area using CD31 immunofluorescence (IF) confirmed a statistically significant increase in the vasculature in response to ELF5 (Fig 2E).

**Fig 3:**
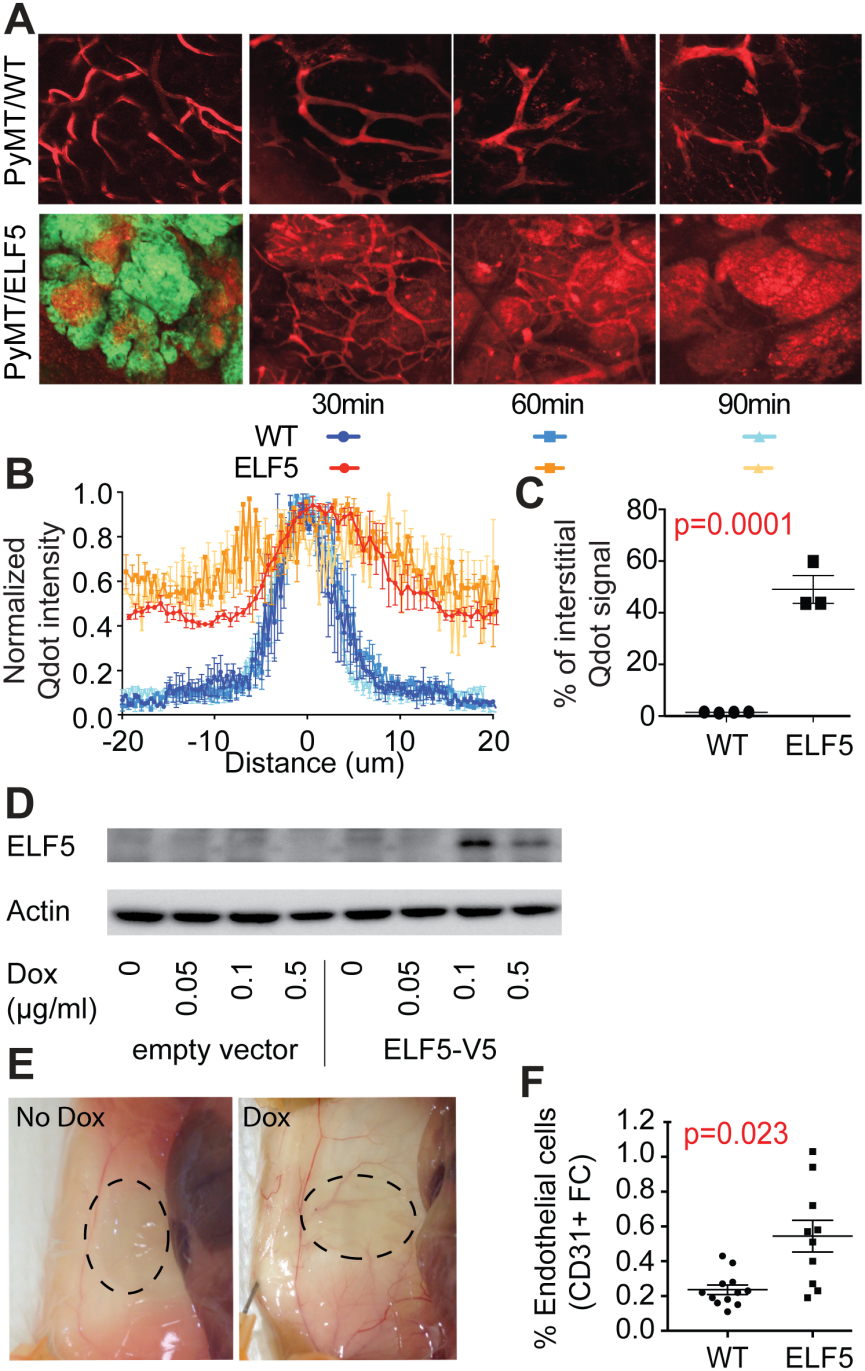
ELF5 drives angiogenesis and the generation of an intra-tumor leaky vasculature. **Panel A**. Intravital real-time microscopy of blood tracer quantum dots (red) injected into the vasculature of mice of the indicated genotypes. LHS panels, quantum dots visualized together with EGFP (green) marking Elf5 expression. RHS panels, imaging of quantum dots (red) in the tumors 30, 60, and 90 min after injection. **Panel B** quantification of quantum dots in relation to the distance from the center of multiple blood vessels in ELF5 animals (red hues) or control animals (blue hues) at the times indicated. Raw data can be found at S2 Data. **Panel C**, statistical analysis of the vascular leakiness revealed by imaging of quantum dots in four and three mice of each genotype, respectively. **Panel D**, induction of ELF5 protein in a PyMT cell line in response to 48 h DOX treatment transduced with the pHUSH DOX-inducible expression vector (PyMT-pHUSH-Elf5) or a pHUSH empty vector. **Panel E**, area occupied by a matrigel plug (indicated by dashes) containing long-term DOX exposed PyMT-pHUSH-Elf5 cells. **Panel F**, endothelial content of the matrigel plugs removed from mice measured by flow cytometry (FC).

We used in vivo real-time intra-vital microscopy to examine tumor vasculature reorganization and increased blood vessel permeability. Intravenous injection of blood tracer quantum dots revealed their accumulation in the interstitial space of PyMT/ELF5 mice treated with DOX for 8 wk (Fig 3A), but not in control animals. Live time course imaging at the times indicated in Fig 3A showed that quantum dots accumulated in the interstitial space within minutes of injection and reached a steady state after 1 h. Quantification showed that accumulation of quantum dots in the spaces beyond 5 um from the center of major vessels was mostly complete within 30 min (Fig 3B). Blood vessel permeability was found to be very consistent between individual mice of the same genotype and the increased permeability of ELF5high tumors was highly statistically significant (Fig 3C).

The ability of Elf5 to induce an angiogenic response in the PyMT tumors was analyzed using an independent experimental system. Two independent cell lines established from explanted PyMT tumors were stably infected with the pHUSH construct encoding a DOX inducible *Elf5* (V5 tagged) expression cassette [16]. PyMT-ELF5-V5 cells robustly expressed ELF5-V5 upon DOX exposure (Fig 3D). PyMT-ELF5-V5 cells were maintained in culture with and without DOX for 2 wk, harvested, re-suspended in matrigel and placed subcutaneously in the flank of congenic FVB/n recipients. Hosts on DOX showed increased recruitment of vasculature around the implantation site (Fig 3E). Flow cytometric analysis of the cells captured within the matrigel revealed greater infiltration of CD31+ cells from DOX treated hosts (Fig 3F).

Overall these data demonstrate that ELF5 exerts a potent angiogenic force that produces an aberrant leaky vasculature.

### Forced Elf5 Expression Induces Metastatic Ability of PyMT Mammary Tumors

**Fig 4:**
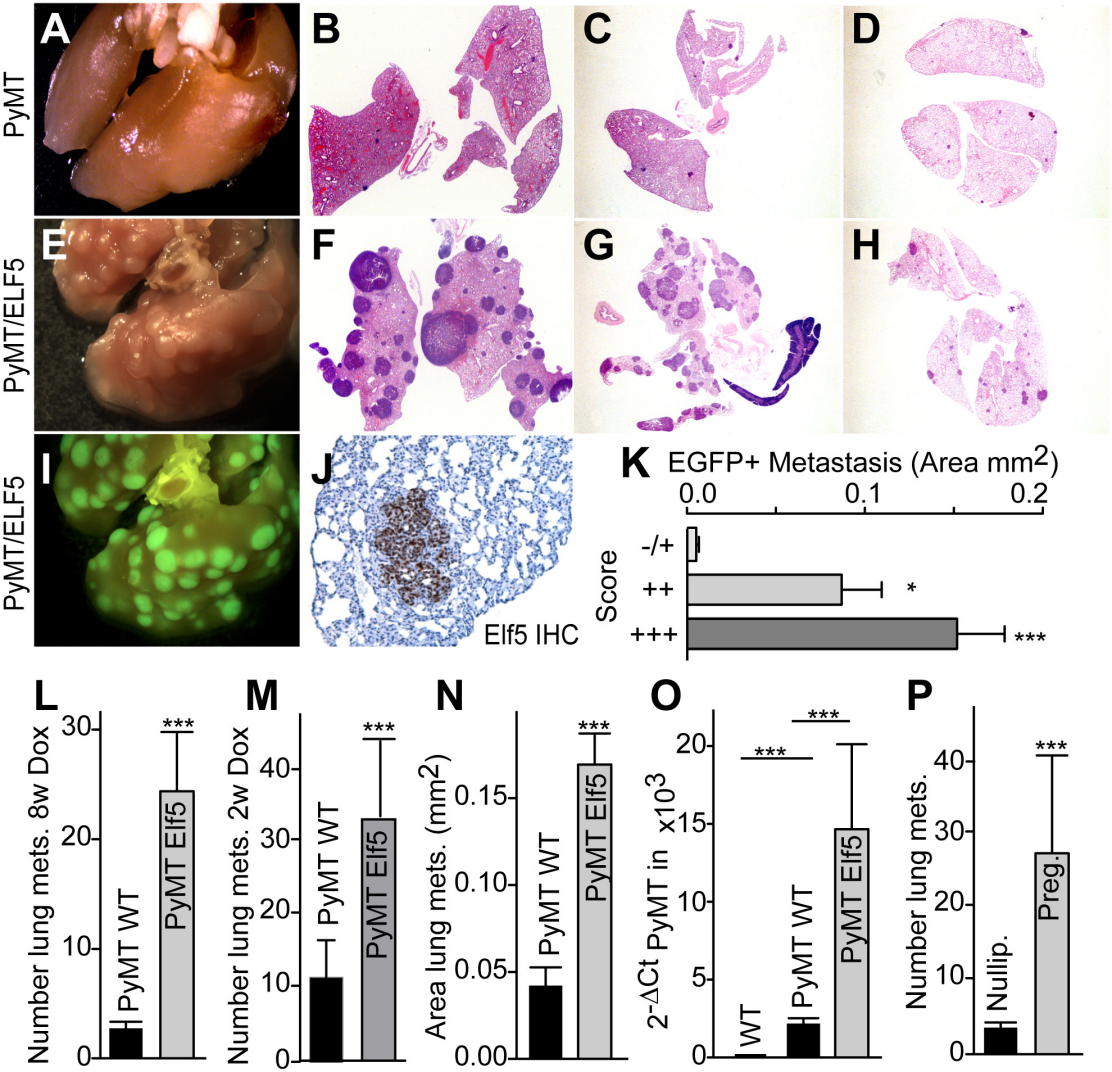
ELF5 expression in the tumor epithelium increases metastases to the lungs. **Panel A**, appearance of lungs from a control PyMT animal following long term (8 wk) DOX treatment. **Panel B**, H&E histology of lungs in Panel A. **Panels C and D**, examples of H&E histology of lungs from control PyMT mice receiving short term (2 wk) DOX treatment. **Panel E**, appearance of lungs following long term ELF5 expression. **Panel F**, H&E histology of the lungs in Panel E. **Panel G and H**, H&E histology of lungs from mice receiving short term induction of ELF5. **Panel I**, visualization of EGFP of the lungs in Panel E. **Panel J**, example of IHC staining for ELF5 in a PyMT/ELF5 lung metastasis. **Panel K**, relationship between the size of an individual lung lesion and the IHC score for ELF5 level (combining intensity and percent positivity). **Panel L and M**, quantification of the number of metastases in the lungs of the mice with the indicated genotypes after long or short term DOX exposure respectively. **Panel N**, metastatic behavior of the indicated genotypes expressed as an area. **Panel O**, PyMT expression measured by qPCR in the blood of mice of the indicated genotypes. **Panel P**, comparison to the number of metastases driven by pregnancy. Labels are pregnancy (preg.) and nulliparous (nullip.). Raw data for panels K, L, M, N, O, and P can be found at S3 Data.

We examined the effect of the induction of ELF5 on the metastatic behavior of the PyMT model. In control animals, constitutive PyMT expression produced no visible lung metastatic nodules by the time the primary tumors reached the ethical endpoint of 10% body weight (Fig 4A), but small metastases within the lungs were detectable by H&E histology (Fig 4B). DOX administration in control animals had no effect on metastasis (Fig 4C and 4D). Induction of ELF5 from 6 wk of age resulted in a dramatic increase in metastasis to the lungs, now visible as numerous nodules on the surface of the lung at the ethical endpoint (Fig 4E) and large and numerous metastases within the lungs by H&E histology (Fig 4F). Induction of ELF5 for 2 wk once tumors were palpable also increased the size and number of detectable lung metastases (Fig 4G and 4H) but with more variable penetrance between animals compared with longer DOX treatment. Most of these metastases expressed ELF5, observed by visualization of EGFP (Fig 4I) and by ELF5 IHC (Fig 4J). Quantification showed a positive correlation between the size of the metastatic lesion and the level of ELF5 protein (Fig 4K). Unlike the primary tumors the metastases showed no regions of hemorrhage. Quantification of H&E stained sections showed statistically significant increases in the number of lung metastases (Fig 4L and 4M). Measurement of metastatic area produced similar results (Fig 4N). Induction of ELF5 greatly increased the amount of PyMT-mRNA present in blood (Fig 4O), suggesting increased numbers of circulating tumor cells. Elf5 is a master regulator of the development and remodeling of the mammary epithelium during pregnancy. During this period Elf5 is intensively expressed. We found that the metastasis-promoting effect of Elf5 was comparable to that produced by pregnancy in this model (Fig 4P).

### Transcriptional Activity of ELF5 in Cancer Cells Drives Inflammation in PyMT-Tumors

**Fig 5:**
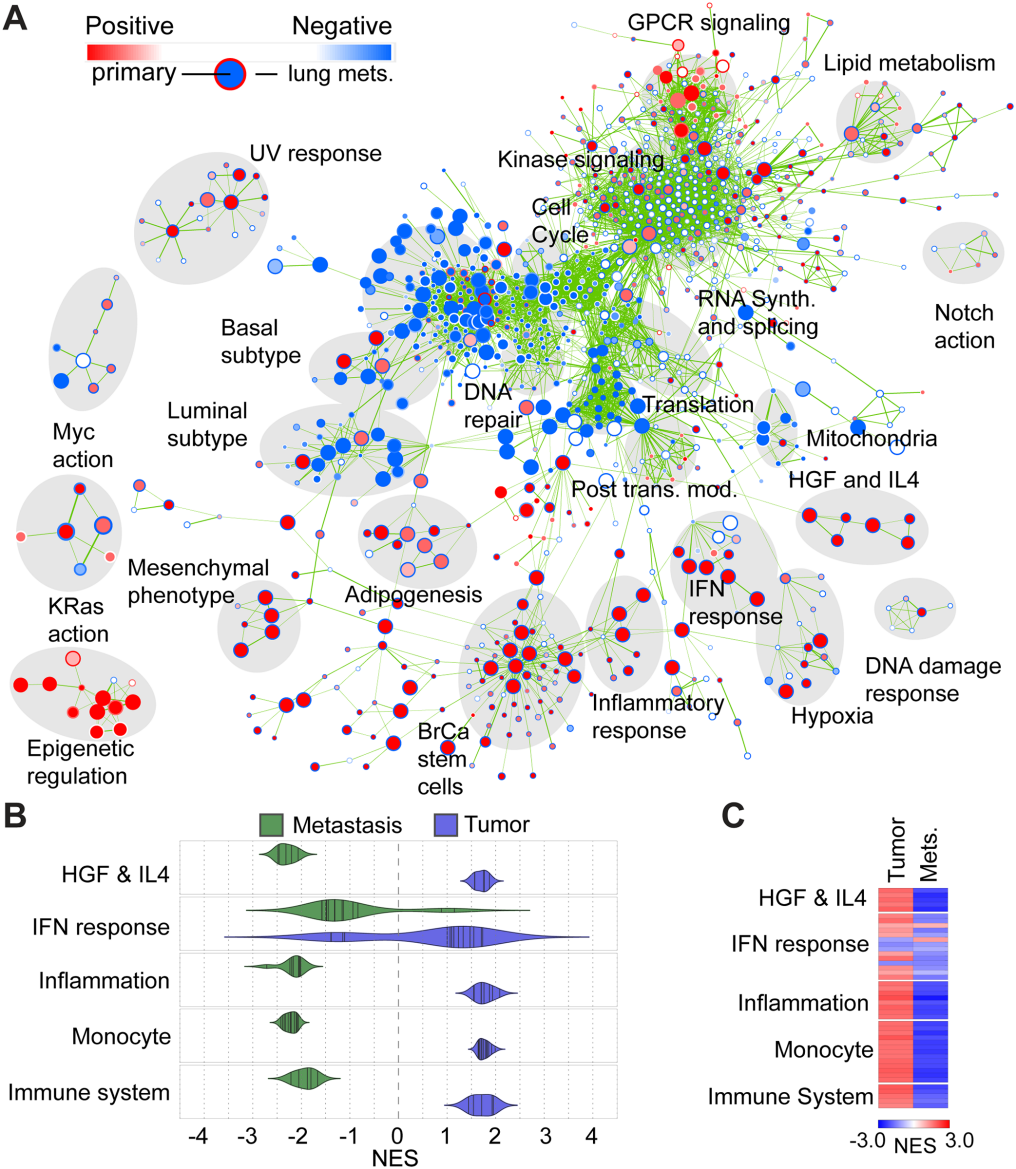
Patterns of gene expression driven by ELF5 in primary tumors, and comparison to subsequent changes in gene expression following metastasis. **Panel A**, Cytoscape Enrichment Map visualization of a gene set enrichment analysis (GSEA) of Affymetrix microarray data comparing EGFP high mammary cancer cells with WT cells from primary tumors (inner node color). EGFP high cell gene expression is then compared to that from EGFP high cells from lung metastasis (outer node color). Labels summarize the functions of gene set clusters indicated by grey shading. Red node color indicates positive gene set enrichment and augmentation of function. Blue node color indicates negative enrichment and the suppression of function. **Panel B**, violin plots comparing GSEA normalized enrichment scores for inflammatory functions in the tumors and their metastases. **Panel C**, heatmap showing normalized enrichment score (NES) score value for each individual gene set included in the defined functional clusters. Gene-set names and statistics can be found in S1 Table.

We purified Lin- CD24+ EGFP+ mammary epithelial cancer cells from the primary tumors and lung metastases of DOX treated PyMT/ELF5 mice, and Lin- CD24+ mammary epithelial cancer cells from the primary tumors of DOX treated PyMT/WT mice, and examined the differential patterns of gene expression using Affymetrix arrays analyzed by LIMMA. Functional gene networks were identified by Gene Set Enrichment Analysis (GSEA) and were visualized using the Enrichment Map plugin for Cytoscape software (Fig 5A) (for a PDF version that can be zoomed in on, see S3 Fig). EGFP+ cells were compared to WT cells from primary cancers to discover functions altered by ELF5 induction, shown by the inner node color, while the outer node color shows how these functions changed in EGFP+ primary compared to EGFP+ lung metastasis. Functions related to cell cycle control, DNA repair, transcription, and translation were suppressed by ELF5 during primary carcinogenesis and remained similarly suppressed in the metastases. Aspects of kinase-based cell signaling were increased by ELF5 during primary carcinogenesis but were then generally suppressed following metastases, although GPCR-mediated signaling increased during carcinogenesis and increased again following metastasis. These results are consistent with Elf5 action in human breast cancer cell lines MCF7 and T47D [16]. Strikingly, we identified functional clusters related to an inflammatory response that were activated in the ELF5-driven primary tumors, but reversed in the metastases. To investigate this further, we extended the GSEA to include molecular signatures of immunologic origin. Guided by an automated clustering approach, we identified gene-sets related to HGF and IL4, inflammation, immune system and interferon responses, and activated monocytes, which were all enriched in the primary tumors in response to ELF5 and suppressed in the metastases (Fig 5B). Fig 5C shows a heat map of the Normalized Enrichment Score (NES) for each individual gene-set included in the defined functional clusters (S1 Table).

We identified patients from the TCGA breast cancer cohort that were classified as having either a luminal A or luminal B PAM50 molecular subtype [34]. Each luminal subtype was stratified on ELF5 expression levels and ranked gene lists of differential expression were generated using LIMMA. These ranked lists were used as the input for GSEA, to allow comparison of the transcriptional response correlated with increased *ELF5* expression in human luminal cancers. We found a positive correlation in luminal A tumors, whereas a negative correlation was found in luminal B patients (S4A and S4B Fig). Higher *ELF5* expression in luminal A, but not B breast cancers, was broadly associated with the same five functional networks identified in the PyMT/Elf5 model: HGF and IL4, invasive phenotype, monocytes, immune system involvement, inflammation and the interferon response (S4C and S4D Fig). These observations suggest that *ELF5* expression produces a more similar response in human luminal A breast cancer to that observed in the ELF5-driven mouse PyMT model.

Taken together, these findings confirm our observations made in human breast cancer cells regarding the function of ELF5, and indicate that, in vivo, these effects are coupled with the immune system, both in the PyMT model and in luminal A human breast cancer.

### ELF5 Drives Metastasis to the Lung through a Mechanism Involving Granulocytic MDSC

**Fig 6:**
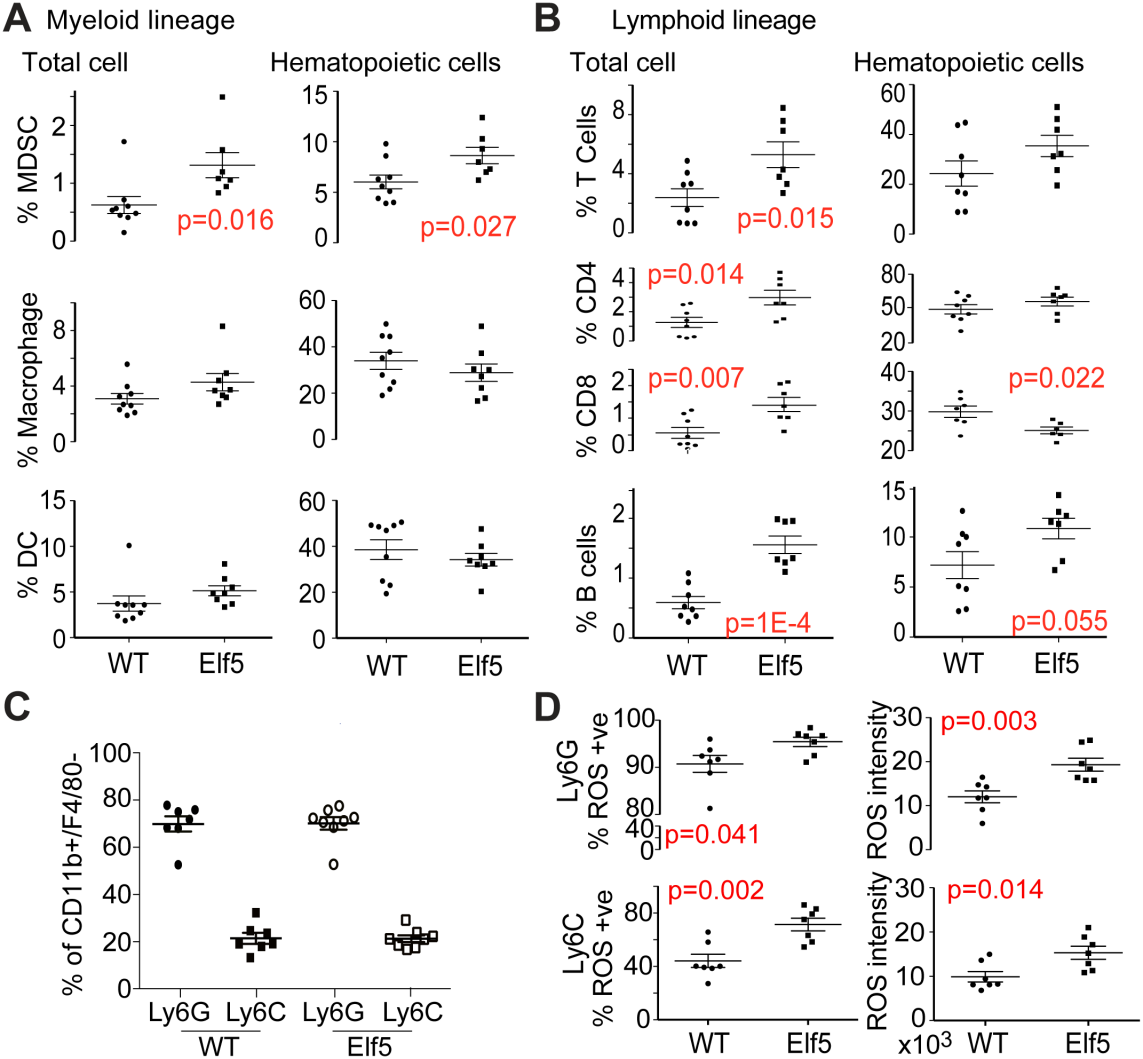
Immune cell subsets within tumors and the effect of depletion of MDSCs on ELF5-driven lung metastases. **Panels A and B**, tumor content of myeloid or lymphoid lineage immune cell subsets expressed as either a proportion of total tumor cells (after erythrocyte lysis) or as a proportion of CD45+ hematopoietic cells. S5 Fig shows the gating strategy. **Panel C**, relative proportions of Ly6G and Ly6C MDSC in mice of the indicated genotypes (Ly6G/Ly6C ratio between genotypes are nonsignificant Student’s *t* test *p* = 0,886). **Panel D**, the effect of ELF5 on the proportion of reactive oxygen species (ROS) positive Ly6C or Ly6G cells (LHS panels) or on ROS cellular intensity measured as geometric mean (RHS panels). Nonsignificant *p*-values are not shown.

We sought to characterize the ELF5-driven inflammatory phenotype and its effect in metastasis. There is an extensive and persuasive literature regarding the pro-angiogenic and -metastatic roles of innate immune cells in the PyMT model. New drugs targeting the immune system are currently revolutionizing cancer treatment. We examined the recruitment and activation of tumor immune cell infiltrates in response to ELF5 using flow cytometry. We measured myeloid (Fig 6A) and lymphoid (Fig 6B) lineages as a percentage of the remaining total cells, or as a proportion of total CD45+ hematopoietic cells. S5 Fig shows the gating strategy and cell surface markers used to produce this analysis. Among the myeloid populations, MDSCs (defined as Gr-1+CD11b+) showed an increased proportion of either total cells or hematopoietic cells, however no significant changes were observed in the number of the other myeloid populations analyzed (Fig 6A). T- and B-cell lymphoid lineages increased as a proportion of total cells, indicative of increased inflammation (Fig 6B). Proportional with the total leukocyte population, B-cell increase was 1.5-fold higher in ELF5 tumors. Within the leukocyte T CD3+ population, T-CD8+ cell number was significantly decreased (2-fold) but no change was observed in the T-CD4+ population, increasing the T-CD4 to -CD8 cell ratio consistent with a MDSC-driven pro-tumorigenic immune suppressive microenvironment.

MDSC (Gr1+) can be subdivided in the granulocytic and the monocytic subset according to their expression of the antigen molecules Ly6G and Ly6C, (Mo-MDSC (CD11b+Ly6G-Ly6Chigh) and G-MDSC (CD11b+Ly6G+Ly6Clow) [35,36]. Flow cytometric analysis of these subsets in PyMT tumors determined that the main population was the Ly6G+ granulocytic subset (Fig 6C). Reactive Oxygen Species (ROS) play a major role in MDSC-mediated immune suppression though the impairment of T cell activation [26]. ROS production by MDSC was significantly increased in both infiltrated granulocytic and monocytic subsets in response to Elf5, consistent with a tumor permissive environment (Fig 6D). A large proportion of the infiltrated Ly6G+ population presented ROS production and this number was further increased to nearly 100% in response of ELF5. The intensity of ROS production was also increased in the MDSC populations in response to ELF5 (Fig 6D). Thus Elf5 increased the number and suppressive ability of tumor-infiltrated MDSC.

**Fig 7:**
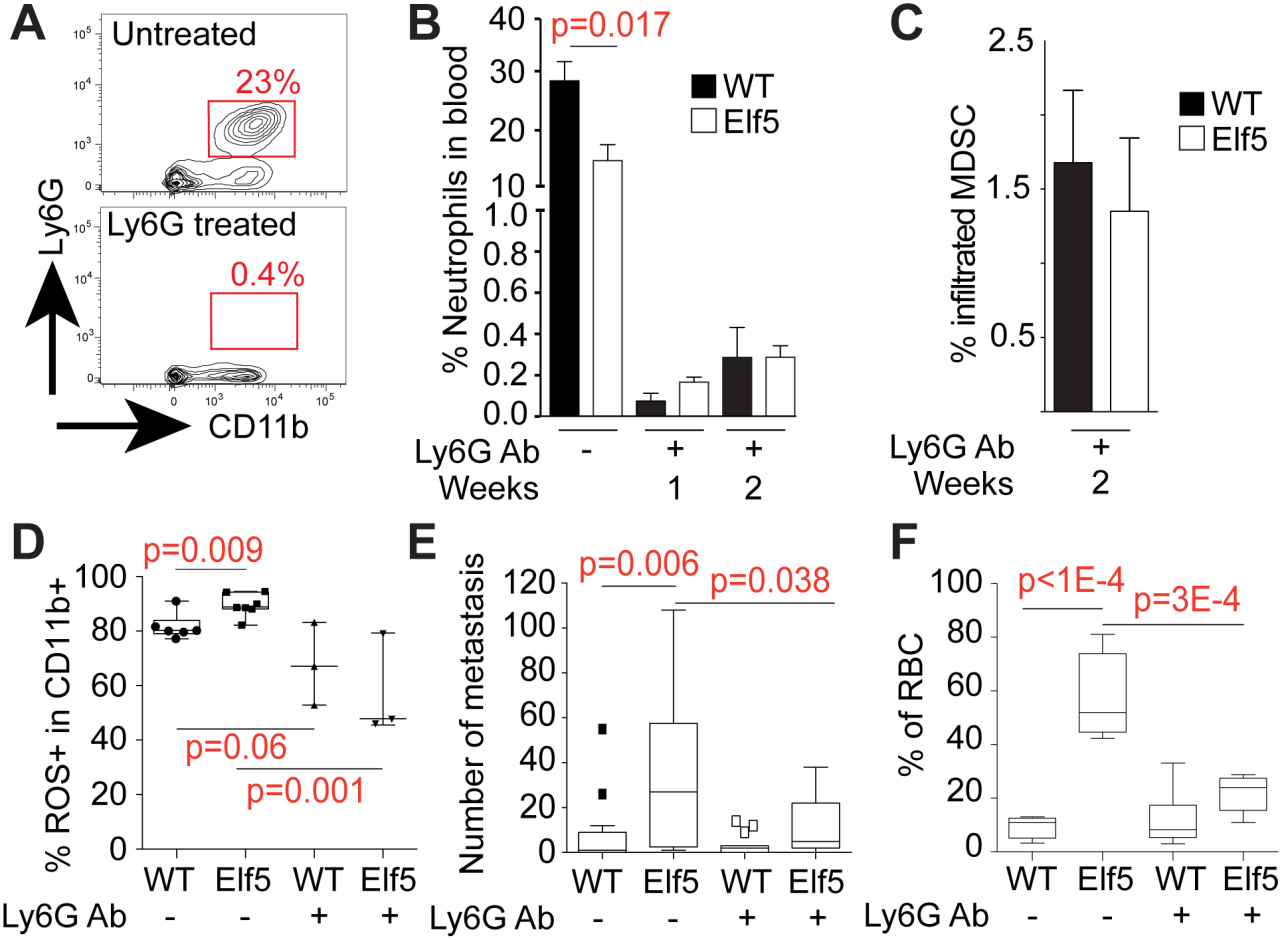
Effect of depletion of MDSC on ELF5-driven lung metastasis and vascular permeability. **Panel A**, representative plot of the depletion of Ly6G+ cells by treatment for 2 wk with the Ly6G antibody. **Panel B**, Time course for the depletion of neutrophils by treatment with the Ly6G antibody. **Panel C**, Effects of Ly6G antibody treatment on tumor infiltrated MDSC in mice of the indicated genotypes. **Panel D**, effect of Ly6G treatment on total ROS production in the CD11b+ myeloid population measured by FACS. **Panel E**, effect of Ly6G treatment on lung metastases. **Panel F**, effects of treatment with the Ly6G antibody on tumor vasculature leakiness. RBC: red blood cells. Raw data for panels B and C can be found at S4 Data.

To determine if the increase in MDSC could account for the increase in metastases caused by ELF5, we used the specific Ly6G antibody to deplete the granulocytic MDSC population during induction of ELF5 in PyMT tumors. Two weeks of treatment with the rat Ly6G antibody resulted in a consistent and efficient depletion, no granulocytic MDSCs were observed in the blood of Ly6G-treated animals (Fig 7A), and a 98% depletion of tumor-infiltrated MDSC was observed (Fig 7B). As a result, only 1.5% of infiltrated Ly6G+ granulocytic MDSC cells were identified in both PyMT/WT and PyMT/ELF5 tumors in the CD11b+ compartment (Fig 7C). Ly6G depletion did not significantly affect the numbers of other infiltrated immune populations in PyMT tumors (S6 Fig). An analysis of the ROS production in the tumor infiltrated CD11b+ myeloid population showed a reduction of total ROS producing cells, consistent with a Ly6G granulocytic cell depletion and a less immune-permissive environment (Fig 7D). MDSC depletion reduced the number of lung metastases in both WT and ELF5 tumors (Fig 7E). We also observed that the antibody treatment reduced the number of red blood cells within the primary tumor (Fig 7F), establishing MDSCs as a key part of the mechanism responsible for both induction of metastases and the hemorrhagic tumor phenotype by ELF5.

### ELF5 Is a Predictive Marker of Poor Prognosis in Breast Cancer

**Fig 8:**
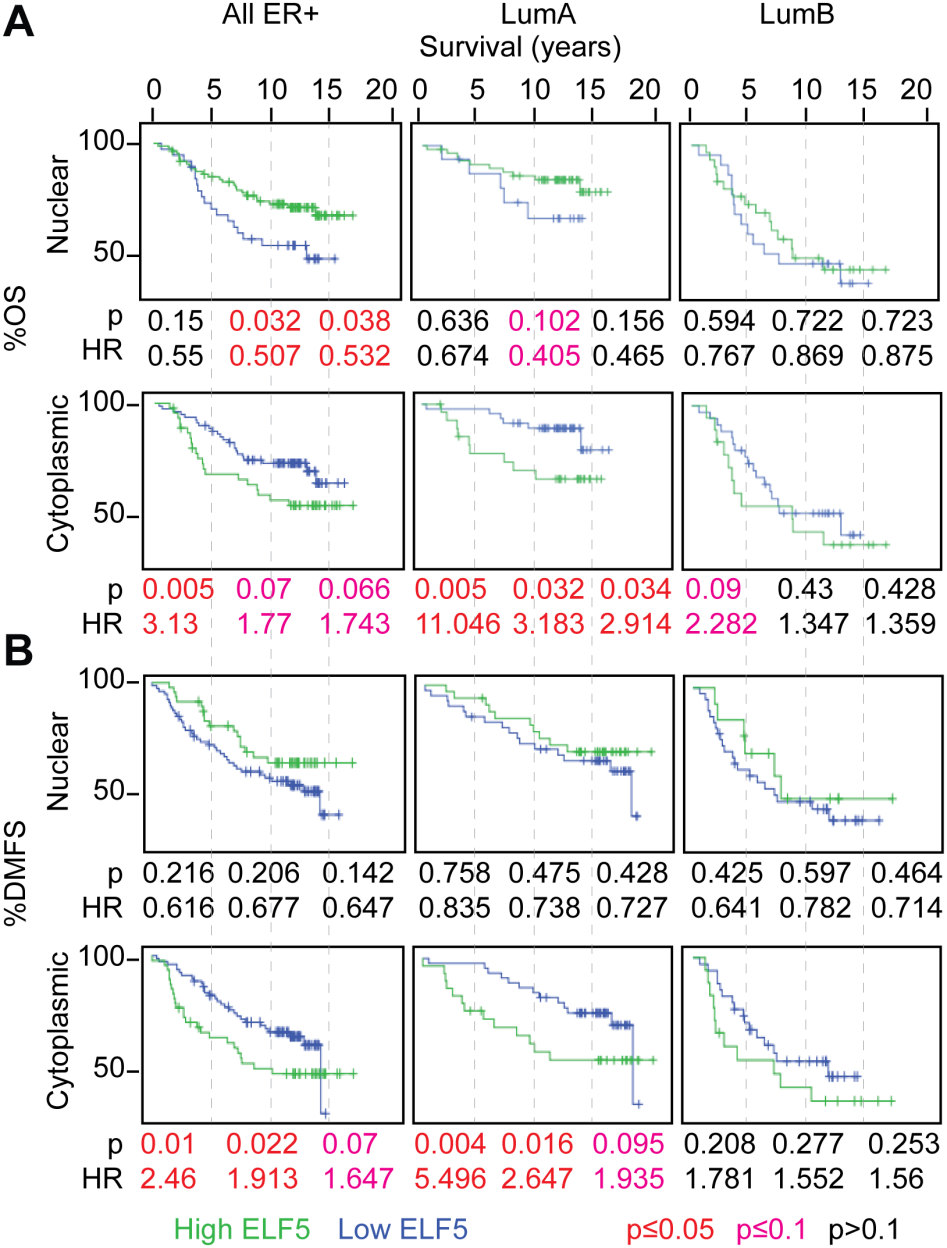
Elf5 immunohistochemistry as a predictor of luminal breast cancer survival. ELF5 was measured by immunohistochemistry in the cytoplasm and nucleus of tumors in a subset of ER+ samples from the Nottingham breast cancer series. **Panel A**, overall survival (OS) and **Panel B** distant metastasis free survival (DMFS). Hazards ratio (HR) and Log Rank *p*-value (p) are given for 5, 10, and 15 y of follow-up. Tumors are split into high ELF5 expression (green) and low Elf5 expression (blue) by XTile and *p*-values are black where >0.1, red where ≤0.05, and pink where 0.05–0.1.

To study the relevance of ELF5 in metastasis in luminal breast cancer patients, we analyzed a cohort of ER+ HER2- tumors staining for ELF5 protein levels using IHC (Figs 8 and S7). This cohort has more than 15 y of clinical follow-up [37]. All patients were treated with the antiestrogen Tamoxifen and none received chemotherapy. We observed nuclear and cytoplasmic patterns of ELF5 staining. Across all ER+ cancers, higher nuclear ELF5 staining predicted better overall survival (OS) after 10 and 15 y but not after 5 y (Fig 8A LHS). This prediction was relatively weak as the hazard ratio was 0.5 and the *p*-value 0.03. In contrast, higher cytoplasmic ELF5 staining predicted worse survival, and at 5 y this prediction was strong, with the hazard ratio greater than 3 at a *p*-value of 0.005. These same effects were evident for distant metastasis free survival (DMFS) where again cytoplasmic ELF5 level was a strong predictor of poor survival (Fig 8B LHS).

We used the St. Gallen definition of Ki67% to split these ER+ cancers into luminal A and B tumors [38]. We found that cytoplasmic ELF5 staining in luminal A patients predicted poorer overall survival with a large hazards ratio, especially at 5 y when it was 11 (Fig 8A). A similar effect was evident for distant metastasis free survival and a large hazard ratio was again evident at 5 y (Fig 8B). Nuclear staining in luminal A patients weakly correlated with poor prognosis in the 10 y follow up overall survival but this prediction was not maintained after 15 y follow up. In contrast, ELF5 levels either cytoplasmic or nuclear, had no predictive value for survival in the luminal B subtype. These results show that ELF5 predicts poorer survival and metastasis in the Luminal A subgroup and that it is a marker of early progression in this subtype.

An interesting observation, given that ELF5 is a nuclear transcription factor, is that cytoplasmic rather than nuclear staining provides this prediction in luminal A breast cancer patients. Although abrogation of ELF5 transcriptional action by restriction to the cytoplasm is suggested by this finding, alternative explanations exist. For example, the antibody epitope may be obscured when ELF5 is bound within a specific transcriptional complex so we caution against over interpretation of the nuclear/cytoplasmic dichotomy until it is better understood.

We studied the immunogenicity of ER+ luminal breast cancer tumors in relation to Elf5. In the absence of a reliable immunohistochemical technique that detects MDSC we instead correlated ELF5 IHC protein levels from this cohort with staining for lymphocytes. We used CD3 and the cytotoxic specific T CD8 maker, the T cell subset targeted by MDSC that was identified in the PyMT/ELF5 model (S2 Table). In this cohort of patients, it has been demonstrated that tumor infiltrated T CD8+ cells correlate with better patient prognosis, suggesting that presence of this cell type is associated with immune tumor rejection [39]. The presence of lymphocytes was analyzed according to their location, intratumoral (within the tumor nests), in the adjacent stroma and in distal stroma. Cytoplasmic ELF5 staining significantly correlated with increased intratumoral T CD3 cell numbers in the luminal ER+ cohort (Spearman’s rank *p* = 0.11, r_s_ = 0.156), with no correlations seen with T cells adjacent or distant to the tumor. Despite this increase in total T lymphocytes in ELF5-high expressing tumors, the number of intratumoral T-CD8+ lymphocytes were significantly underrepresented (Spearman’s rank *p* = 0.04, r_s_ = -0.203). Categorical Mann-Whitney analysis (cut off CD3 ≥ 2 cells; CD8 > 1 cell, based on X-tile analysis) confirmed the direct association (*p* = 0.075) between cytoplasmic ELF5 expression and intratumoral CD3 infiltration and the negative correlation with the T-CD8+ subset (*p* = 0.046). When the ER+ cohort was split into luminal A and B subtypes these effects were maintained, although the statistical power of the analysis was reduced due to the sample number (S2 Table). Interestingly, a strong inverse association of nuclear ELF5 staining and T-CD8+ cells was identified in the ER+ cohort and in the Luminal B subgroup.

These data indicate that luminal ER+ tumors with high Elf5 levels show higher intratumoral T lymphocytes, however the cytotoxic T-CD8+ population is selectively reduced. Our results in human breast cancer are consistent with our observations in mice suggesting the implication of ELF5 in a tumor permissive inflammatory environment. These data establish a strong case for further investigation of the role played by Elf5 in immunosupression and its relationship with survival in luminal A breast cancer.

B-cell lymphocytes analysis using the B20 marker in the Nottingham cohort revealed a high number of samples with absent staining [40]. Fifty-six percent (73/130) of the cases in this study were completely negative for B20 and 80% (105/130) lay below the statistical x-tile cutoff (B20 > 5 cells). In the positive cases, B-cells infiltrated in the tumor nests were rare, with the majority of B-cells localized at the distal stroma. No correlation with ELF5 expression was found using intratumoral or adjacent stromal B-cell numbers. Spearman and Mann Whitney analysis on total and distal B-cell number revealed inverse associations between ELF5 expression and the CD20 marker as indicated in S3 Table. B-cell number is directly associated with breast cancer specific survival and longer disease free interval in ER+ patients treated with anti-estrogen therapy [40]. In the MMTV-PyMT model, ELF5 contributes to tumor progression; this discrepancy might be as a result of the poor modeling of the distal stroma in the PyMT tumor FACS analysis, where the majority of the tissue analyzed corresponds to intratumoral and adjacent stroma. Taken together, these results indicate that B-cell analysis does not model ELF5 action in luminal breast cancer.

## Discussion

We show that induction of ELF5 in the PyMT model leads to an increase in lung metastasis because ELF5 recruits MDSCs to the tumor, which promotes leaky vasculature and causes an increase in lung metastasis. Interestingly this effect swamps the cell autonomous effects of ELF5, which predict a tumor suppressor action. Analysis of human breast tumor data suggests that these processes also operate in ER+ breast cancer, and analysis of survival data shows this is prognostic in Luminal A cancers, with ELF5 expression in the cytoplasm clearly identifying a group of luminal A patients with early disease progression. High cytoplasmic ELF5 expression in luminal patients also correlated with a pro-tumor inflammation characterized by decreased cytotoxic T-CD8 lymphocytes.

*ELF5* has been proposed by Chakrabati and colleagues as a metastasis suppressor gene for all breast cancers [18], but our studies demonstrate that the luminal A subgroup shows the opposite response. Interestingly, we show that ELF5 produces a number of cell-autonomous phenotypic changes that are consistent with a tumor-suppressor role, such as reduced proliferation, invasion, motility, epithelialization, and colonization in a lung-seeding assay, some features of which have been previously reported by us [16] and by Chakrabati and colleagues [18] using different model systems. Our results point to the dominance of the immune system over cell autonomous characteristics in regulating the metastatic behavior of luminal A primary tumors, and so to the importance of pursuing immunoregulatory therapies for luminal A breast cancer.

Given the previously described role of ELF5 in the progression to antiestrogen insensitivity in luminal breast cancer, where ELF5 levels rise [16], our results now show that this escape pathway is likely to lead to metastasis via attraction of the innate immune system. This may represent a normal biological response, as macrophages and neutrophils are attracted to the mammary gland during periods of tissue remodeling, especially during weaning when the mammary alveoli are largely resorbed, returning the gland to a series of branched ducts. We observed enrichment of involution and lactation signatures in our transcriptional data in response to ELF5 in both the mouse model and the TCGA data sets. Higher ELF5 expression may result in the tumor being seen by the host as an involuting mammary gland, and the luminal A subgroup may possess a background phenotype which allows or best expresses this appearance. When we treated our mice with the anti MDSC antibody Ly6G we did not completely ablate metastasis, rather we returned metastasis to control levels. This shows other prometastatic pathways continue to operate. One key pathway demonstrated in the PyMT model is the role of macrophages [41], whose numbers were unaffected by ELF5 expression.

Hemorrhagic necrosis and intratumoral hemorrhage is observed in breast cancer [42], where it generates pain due to mastodynia in otherwise painless cancers. Short-term induction of ELF5 in the mouse provides a good representation of this human pathology, where isolated hemorrhagic regions are seen. Longer term induction produces a more severe effect than seen in the clinic. The basis for hemorrhage involves the recruitment of MDSC, as shown by its reduction following suppression of these cells with Ly6G antibody. We speculate that the earlier detection of in situ ELF5 tumors is due to the immune cell infiltration, making them larger than the WT controls, since further monitoring showed that they expanded more slowly. Unlike the primary tumor, our data show that colonies of cells growing in the lungs have found a supportive environment. Transcriptional signatures indicative of cellular stress are lost. Necrotic areas are not present and the hemorrhagic phenotype is lost. Interestingly innate immune system recruitment also appears to be absent in the metastases.

These results indicate that ELF5 is a major determinate of the lethal phenotype in luminal A breast cancer. Elf5 expression provides a marker that defines early disease progression in this otherwise slow to progress subtype, and may also define a group that should benefit from future immunomodulatory therapies.

## Materials and Methods

### Ethics Statement

Mice were maintained following the Australian code of practice for the care and use of animals for scientific purposes observed by the Garvan Institute of Medical Research/St. Vincent's Hospital Animal Ethics Committee (AEC), AEC#11/35 (previous) and AEC# 14/27 (current). Euthanasia was performed by asphyxiation with carbon dioxide gas, followed by cervical dislocation, in a separate area away from other animals. For all surgical procedures, animals were anesthetized with Isoflurane at a rate of 1L/minute oxygen 5% Isoflurane for induction and 1L/minute 2% Isoflurane for maintenance. Animals recovered from surgery at room temperature in a box “half on/half off” over a warm heat pad to prevent hypothermia. They received analgesia systemically and locally. Animals were closely monitored until they had regained the ability to right themselves, then placed individually in cages in a special purpose room. When required, animals were checked for blood on their coats that will be removed before they wake up from anesthesia. The next day animals are checked for general condition (e.g., alertness, weight loss, balance, and mobility).

### Experimental Animal Models

The Elf5 inducible PyMT mammary tumor transgenic model has been generated by crossing the MMTV- Polyoma Middle T antigen (PyMT) mouse mammary tumor model [30] with the doxocyclin (DOX) inducible Elf5 Knock In mouse line [8]. The inducible promoter induces a bicistronic cassette codifying for the human version of Elf5 followed by EGFP using an IRES sequence. We used the rtTA locus under the MMTV promoter to control the expression of Elf5 in the mammary epithelial cells (MTB animals). All animals used in this study are heterozygous for Elf5, MTB, and PyMT. S1A Fig shows a schematic representation of the transgenic cassettes and genotypes used for the study. To induce the expression of the Elf5 and EGFP mice were exposed to a diet containing 700 mg/Kg of Doxocyclin (Gordon’s Specialty Stockfeeds). For the neutrophil depletion experiment, 100 μg of Ly6G antibody clone 1A8 (UCSF) was injected IP twice a week for 2 wk, a pretreatment injection was performed 2–3 d before DOX exposure. Syngenic FVB/n hosts were used for matrigel plug assays.

### Cells and Constructs

Elf5 was tagged at the 3′ end with V5 and incorporated into the pHUSH-ProEX vector (Genentech) [43] as descried before [16]. Elf5 expression was achieved using Doxycycline (Clontech) at 0.1 μg/ml. Luciferase/GFP [44] and pHUSH-ProEx plasmids were packed into retrovirus using PlatinumE cells (Cell Biolabs) using FuGene6 or X-Treme transfection reagent (Roche) following manufacturer instructions. PyMT cell lines were established in culture from enzymatically disaggregated PyMT tumors and double FACS-purification based on CD24 expression; and were maintained in DMEM medium containing 10%FBS, 1% L-Glutamine, 5 ug/ml Insulin, EGF 10 ng/ml, and 10 ng/ml cholera toxin. The line was considered to be established in culture after ten passages.

### Flow Cytometry and Antibodies

Flow cytometry was performed using FACS Canto II or LSR II (analysis) and FACS Aria III (analysis and sorting) from Becton Dickinson and exported to the FlowJo software (Tree Star Inc.) for data analysis. Reactive Oxygen Species was measured using the DCFDA reagent (Abcam). DAPI ([4′,6-diamidino-2-phenylindole dihydrochloride]) (Molecular Probes) or Propidium Iodide (Sigma) was used as death cell exclusion marker. Flow cytometry was performed using the following fluorophore conjugated antibodies: CD45, CD31, Ter119 from BD Pharmingen; CD3 (clone 17A2), F4/80 (clone BM8), Gr-1 (clone RB6-8C5), CD4 (clone GK1.5), CD8 (clone53-6.7), CD11c (clone N418), CD11b (clone M1/70), and B220 (clone RA3-6B2) from eBioscience; Ly6G (clone 1A8) and Ly6C (clone HK1.4) antibodies were purchased from BioLegend. For neutrophil depletion experiments Ly6G antibody (clone 1A8) was used (UCSF or Bio X Cell) and FACS performed using an anti-rat IgG secondary form BioLegend. A list of the defined populations using these antibodies is listed in S4A Fig. IF for CD31 was performed using OCT embedded tissue and the BD Pharmigen antibody clone MEC13.3.

### Matrigel Plug Assays

Two established PyMT cell lines were stably transduced with a DOX-inducible pHUSH vector encoding Elf5 tagged with the V5 peptide [16]. PyMT pHUSH-Elf5-V5 cells were then exposed to 0.1 μg of DOX every other day for 10 d or remained untreated for control. 10^5^ long term DOX and control PyMT pHUSH-Elf5-V5 cells were then harvested and mixed with 4C matrigel (1:9/vol:vol) and immediately injected subcutaneously in the flank of FVB/n recipients. Hosts were exposed to DOX containing food 24 h prior matrigel implantation and until collection or left untreated for control cells. Ten days after implantation matrigel plugs were extracted, cell suspensions prepared using collagenase digestion and processed for FACS analysis.

### Gene Expression Microarray Profiling Analysis

Normalization and probe set summarization was performed using the robust multichip average [45] implemented in the Affymetrix library [46] from R [47] as part of the *NormalizeAffymetrixST* module in GenePattern. Control probe-sets were removed from the arrays. Differential gene expression was then assessed for each microarray probe set using an empirical Bayes, moderated t-statistic implemented in Limma (Smyth, 2004) using the *limmaGP* tool in GenePattern. All pairwise experimental comparisons performed are described, where relevant, in the text.

Where indicated, the analysis tools utilizing GenePattern software [48] are available at the Garvan hosted GenePattern server http://pwbc.garvan.unsw.edu.au/gp/. Microarray data are available from GEO: GSE58729. Detailed information about mRNA extraction, purification, chip hybridization and processing can be also found in this link. All analysis results, additional GSEA gene-sets, and custom analysis scripts are available on request from the authors.

### The Cancer Genome Atlas Database

For the analysis of TCGA expression data, clinical and molecular annotation of samples was obtained from the Cancer Genome Atlas (TCGA) breast cancer publication [49]. Agilent mRNA expression microarray data (Level 3) was obtained from the TCGA data portal in January 2012. Missing expression values were imputed and replaced using the k-nearest neighbor (KNN) approach, with k = 10 (using the *ImputeMissingValuesKNN* module in GenePattern). The TCGA microarray data consisted of a total of 533 tumors. From this, we generated 2 subsets of patients based on their PAM50 classified molecular subtype [34], 231 with a Luminal A PAM50 sub-type and 127 with a Luminal B PAM50 subtype.

The samples in each of these luminal patient subsets, were each stratified on expression level of ELF5, and the top 25% (ELF5^hi^) and bottom 25% (ELF5^lo^) expressing samples were selected. For each of these ELF5 stratified groups, differential gene expression between ELF5^hi^ and ELF5^lo^ patient groups was assessed, for each gene, using an empirical Bayes, moderated t-statistic implemented in LIMMA [50] via the *GP* tool in GenePattern.

### Gene Set Enrichment Analysis (GSEA)

For all pair-wise experimental comparisons, Gene Set Enrichment Analysis (GSEA) [51] was run in pre-ranked mode using a ranked list of the LIMMA moderated t-statistics. One thousand gene-set permutations were performed using minimum and maximum gene-set sizes of 15 and 1,500, respectively. Gene-sets used in GSEA were extracted from version 3.1 and 4.0 of the Broad institute’s Molecular Signatures Database (MSigDB) [52] and extended with additional curated gene-sets from literature. All GSEA analysis was performed using a combined set of the c2, c6 (for Fig 5A), and extended with c7 gene-sets (for Fig 5B and 5C and S4 Fig) from MSigDB plus additional curated sets that we identified in the literature. This resulted in a total of 5,145 gene-sets (MSigDB v3.1 c2, c6 collections plus custom sets) used in the initial, exploratory analysis, shown in Fig 5A, and an expanded gene-set collection of 6,947 gene-sets (MSigDB v4.0 c2, c6, c7 collections plus custom sets) used in the analysis described in Fig 5B and 5C, and S4 Fig.

Network-based visualization and analysis of the GSEA results was carried out using the *Cytoscape* [53] *Enrichment Map* [54] plug-in, with permissive thresholds of: FDR (Q-value) = 0.25; *p*-value = 0.05 and overlap coefficient cutoff = 0.5. The functional networks definitions were based on the cytoscape pre-annotated clusters tool.

To identify functional clusters of gene-sets that were enriched in the PyMT/ELF5 tumors and the TCGA luminal A ELF5^hi^ tumors an automated clustering approach was used. First, an EnrichmentMap network of the GSEA results of these two comparisons was carried out using conservative thresholds of: FDR (Q-value) = 0.05; *p*-value = 0.001, and overlap coefficient cutoff = 0.5. The “annotate clusters” feature in EnrichmentMap v2.1.0 (build 522) was then used, with default “clusterMaker” MCL cluster parameters, to generate a list of gene-set clusters with two or more members. Guided by these automated clusters and those identified in the exploratory analysis in Fig 5A, we defined five gene-set clusters of functional interest. These are listed in S1 Table along with the associated GSEA statistics.

### RNA and PCR

RNA extraction was performed using the RNeasy extraction kit (Qiagen) following manufacturers procedure. For blood samples, Trizol (Ambion, Life technologies) lysis was performed before kit purification. High-Capacity cDNA Reverse Transcription Kit (Applied Biosystems) was used for the cDNA preparation. Quantitative PCR was performed using the LightCycler480 (Roche) using SYTO9 as a dye and the 2^-ΔCt^ method to analyze expression difference [55]. Q-PCR PyMT in blood was detected using the following primers: Fwd: tgtgcacagcgtgtataatcc and Rv: tcatcgtgtagtggactgtgg; and confirmed with Fwd: taagaaggctacatgcggatgggt and Rv: ggcacctggcatcacatttgtctt; and housekeeping gene GAPD using the following primers Fwd: agcttgtcatcaacgggaag; and Rv: tttgatgttagtggggtctcg. Q-PCR for Elf5 was detected using Taqman probe Mm00468732_m1 or Hs01063022_m1 (Applied Biosystems), and housekeeping gene GAPD, Mm99999915_g1 or Hs99999905_m1; using the 7900H Fast Real-Time PCR system (Applied Biosystems).

### Immunohistochemistry and Immunoblot

For ELF5 and GFP immunohistochemistry, slides were blocked with protein block after antigen retrieval using Dako buffers (pH 6.1 at 125°C for 2 min, or pH9 at 100C for 25 min), followed with 0.05%Tween in PBS or 0.2% TritonX100. Primary antibodies were incubated for 1 h, ELF5 1:500 (N20, sc-9645, Santa Cruz) or GFP 1:200 (A11122, Invitrogen), then followed by either Rabbit anti-Goat 1:100 (Invitrogen) and LSAB+ label (Dako) or Envision Rabbit 30 min (Dako), then detection with DAB+ (Dako). For ELF5 IHC in patient samples, following blocking of the 4 micron paraffin-embedded sections from breast cancer TMAs for endogenous peroxidases, antigen retrieval was performed using pressure cook-microwaving in EDTA buffer (pH 9) for 5 min. This was followed by 0.02% Tween in PBS blocking for 5 min. Primary antibodies were incubated overnight with ELF5 antibody 1:70 in 0.1% BSA.PBS (N20 sc-9645, Santa Cruz) at room temperature. Detection was performed using 1:1000 Rabbit anti-Goat in 0.1% BSA.PBS (Invitrogen A10537) for 20 min, followed by Envison+ system-HRP labelled polymer anti-rabbit for 20 min (Dako 4003). DAB chromogen solution (Dako) was applied for 6 min followed by methyl green counterstaining. ELF5 nuclear and cytoplasmic staining assessment was performed using H-Score analysis that encompasses both percentage positivity and staining intensity on a 0–300 scale.

GFP and BrdU co-immunofluorescence antigen retrieval was pH 9 and 100°C for 25 min, followed by 0.2% TritonX100 then 1:250 GFP (A11122, Invitrogen), and 1:200 BrdU (M0744, Dako) at 4°C overnight. This was followed by 30min incubation with AlexaFluor 488-tagged anti-rabbit antibody, AlexaFluor 555-tagged anti-mouse antibody (1:200; Invitrogen) and ToPro (1:2000; Invitrogen).

Protein analyses by Western Blot were done as previously described [16]. Primary Antibodies used were anti-β-actin (AC-15, Sigma), anti-ELF5 (N20, sc-9645, Santa Cruz) and anti-V5 (R960-25, Invitrogen).

### In Vitro Invasion Assays

Boyden Chamber assays (Bencton Dickinson) were performed by plating 1x10^5^ cells (PyMT) in media containing 0.5% FBS, the chemotactic gradient was established by placing the insets into full media (10%FBS) containing wells. Invading cells were visualized with the Diff Quick Stain Kit (Lab Aids). Area measured with Image J 1.41 (Wayne Rasband, US National Institutes of Health).

### Multiphoton Imaging

Imaging was conducted on an inverted Leica SP8 confocal microscope and the excitation source used was a Ti:Sapphire femtosecond pulsed laser (Coherent Chameleon Ultra II), operating at 80 MHz and tuned to a wavelength of 920 nm. 10 ul of blood tracer quantum dots blood tracers (655nm Life Technologies) were injected through the tail vein of the animals. Images were acquired with a 25x NA0.95 water objective. A dichroic filter (560 nm) was used to separate the GFP signal from quantum dot emission, which were further selected with band pass filters (525/50 and 617/73, respectively). Intensity was recorded with external RLD HyD detectors. For z-stacks, images were acquired at a format of 1,080 × 1,080 and a z-step size of 2.52 μm.

### Statistical Analyses

Sample comparisons have been made by unpaired Student’s *t* test using the GraphPad Prism software, La Jolla California USA. All error bars showed in this paper correspond to standard error (SEM) unless otherwise stated. All analysis in clinical samples were performed using the SPSS software (SPSS Inc. Chicago USA), assessment of the correlation between IHC markers was performed using Spearman rank order correlation and Mann-Whitney U test. Kaplan-Meier curves and log-rank test were used for survival analyses.

### Patient Samples Description

The patient cohort is a subset of the Nottingham series [37] comprising Luminal ER+ patients treated with tamoxifen but no chemotherapy, the distinction of luminal A or luminal B subtype was made according to the St Gallen criteria: *n* = 126 versus survival (74 luminal A, 52 luminal B); *n* = 129 versus DMFS (76 luminal A, 53 luminal B). Optimal staining cutpoints for analysis were selected using Xtile.

## Supporting Information

S1 Data Raw data for Fig 1, panels B, G, and H. (XLSX)

S2 Data Raw data for Fig 3B. (XLSX)

S3 Data Raw data for Fig 4, panels K to P. (XLSX)

S4 Data Raw data for Fig 7, panels B and C. (XLSX)

S1 Fig The ELF5/PyMT mouse model. **Panel A**, schematic representation of the inserted transgenes. The promoter from the mouse mammary tumor virus (pMMTV) drives expression of the reverse tetracycline transactivator (rtTA), which binds doxycycline to activate the tetracycyline-on promoter (pTetOn). This drives expression of a single mRNA encoding ELF5 and the enhanced Green Fluorescent Protein (EGFP), translated as 2 independent proteins by the presence of an internal ribosome entry site (IRES). The Polyoma Middle T (PyMT) oncogene is constitutively expressed from pMMTV. All alleles are integrated separately in the mouse genome. **Panel B**, ELF5 levels in response to DOX administration measured by Western blot, nsb, nonspecific band. DOX was administered either short- or long-tem as indicated.
(TIF)

S2 Fig Effects of ELF5 in tumor growth and cell proliferation. **Panel A**, survival analysis of animals carrying tumors that developed from intraductal transplantation of EGFP+ tumor cells made fluorescent by 7 d administration of DOX, then withdrawing DOX as indicated. The ELF5 transgenic cassette is not selective of a specific epithelial population during tumor progression showed by survival analysis. **Panel B**, proliferation after 7 d DOX treatment measured by BrdU incorporation (red cells) in EGFP high (bright green) compared to EGFP low/no areas (dark green) of primary tumors, quantified by counting cells in random fields (bar chart).
(TIF)

S3 Fig GSEA representation of gene expression changes produced by expression of ELF5. Figure can be viewed at a range of high magnifications, 1,600% or higher, to identify individual gene sets and to see the composition of functional clusters.
(PDF)

S4 Fig Functions correlated with *ELF5* expression in the TCGA series of luminal breast cancers. Differential gene expression associated with *ELF5* expression in PAM50 defined Luminal A and B breast cancer was calculated and ranked (by LIMMA moderated t-statistic) and used as input for GSEA. **Panel A**, shows the Pearson correlation matrix between the normalized enrichment scores (NES) for all gene-sets. **Panel B**, heatmap of the full GSEA-derived transcriptome for Elf5 action in each luminal subtype of the TCGA series compared with the PyMT model, where each row represents the NES of a gene-set and are sorted by PyMT/ELF5 NES. **Panel B**, comparison of the defined inflammatory functional networks by GSEA enrichment scores in each luminal subtype of the TCGA series compared with the PyMT model. **Panel C**, heatmap showing the NES for each individual gene set included in the defined functional clusters. Gene-set names and statistics can be found in S1 Table.
(TIF)

S5 Fig Gating strategy used to isolate MDSCs and other immune cell subsets from PyMT tumors. **Panel A**, definition of the cell sets used in this analysis. **Panel B**, gating strategy. Color coding of antibodies from panel A shows the gated populations they selected.
(TIF)

S6 Fig Ly6G antibody treatment specifically targets granulocytic MDSC within the tumor infiltrated immune populations. FACS analysis of immune infiltrates in tumors from PyMT/WT mice after Ly6G antibody treatment. **Panel A**, shows total leukocytes; **Panel B**, myeloid lineage; and **Panel C** T lymphocytes.
(TIF)

S7 Fig Cytoplasmic and nuclear ELF5 staining. **Panel A**, correlation between cytoplasmic and nuclear staining in the analyzed patient cohort. **Panel B**, prognostic value (OS, overall survival and DMFS, distal metastasis free survival) of the combined cytoplasmic and nuclear ELF5 staining. **Panel C**, prognostic value in the samples positive for nuclear staining only.
(TIF)

S1 Table Gene sets corresponding to the functional clusters defined by GSEA and guided by the automated cytoscape cluster tool. (XLSX)

S2 Table Correlations between ELF5 and the indicated lymphocyte marker within ER+ cancers from the Nottingham cohort using the indicated statistical test. Darker highlight represent stronger statistical association (*p* ≤ 0.05 dark highlight; *p* ≤ 0.1 light highlight) green indicates a negative correlation and red a direct correlation.
(XLSX)

S3 Table Correlations between ELF5 and the B20 lymphocyte marker within ER+ cancers from the Nottingham cohort using the indicated statistical test. Darker highlight represent stronger statistical association (*p* ≤ 0.05 dark highlight; p≤0.1 light highlight) green indicates a negative correlation and red a direct correlation.
(XLSX)

## Abbreviations

DMFS: distant metastasis free survival
DOX: doxycycline
ER: estrogen receptor
FACS: Fluorescence-Activated Cell Sorting
FC: Flow cytometric
GSEA: Gene Set Enrichment Analysis
H&E: haematoxylin and eosin
IF: immunofluorescence
IHC: immunohistochemistry
LHS: left-hand side
MDSC: myeloid-derived suppressor cells
MMP: Matrix Metaloproteinases
MMTV: mouse mammary tumor virus
NES: Normalized Enrichment Score
OS: overall survival
PyMT: Polyoma Middle T
RHS: right-hand side
ROS: Reactive Oxygen Species
VEGF: Vascular Endothelial Growth Factor
WT: wild type
