# Supplementary figures and images for: ELF5 Drives Lung Metastasis in Luminal Breast Cancer through Recruitment of Gr1+ CD11b+ Myeloid-Derived Suppressor Cells
Source: PLoS Biol. 2015 Dec 30;13(12):e1002330. doi: 10.1371/journal.pbio.1002330 (PMC4696735; doi:10.1371/journal.pbio.1002330)

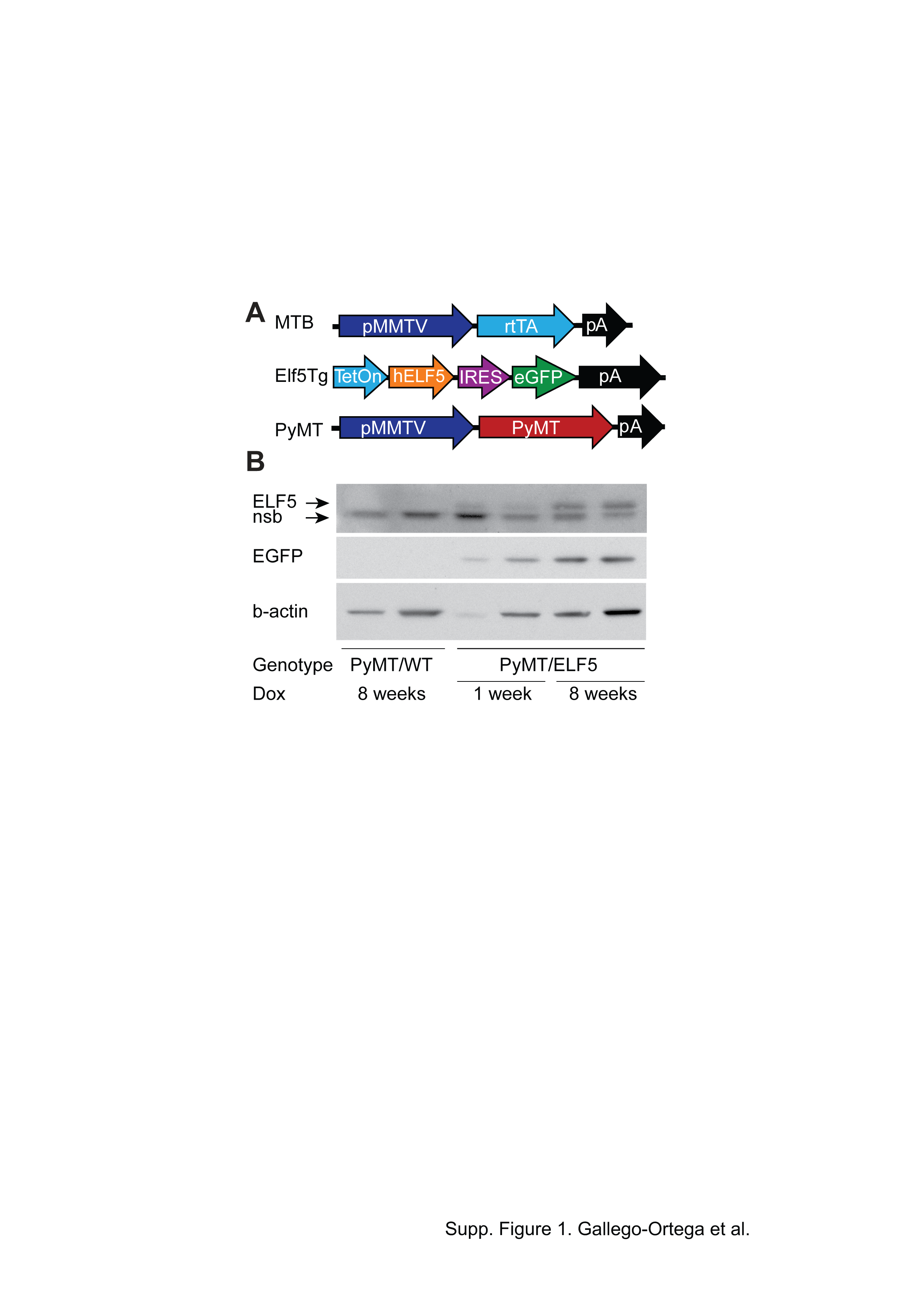

Supplement: S1 Fig — Panel A, schematic representation of the inserted transgenes. The promoter from the mouse mammary tumor virus (pMMTV) drives expression of the reverse tetracycline transactivator (rtTA), which binds doxycycline to activate the tetracycyline-on promoter (pTetOn). This drives expression of a single mRNA encoding ELF5 and the enhanced Green Fluorescent Protein (EGFP), translated as 2 independent proteins by the presence of an internal ribosome entry site (IRES). The Polyoma Middle T (PyMT) oncogene is constitutively expressed from pMMTV. All alleles are integrated separately in the mouse genome. Panel B, ELF5 levels in response to DOX administration measured by Western blot, nsb, nonspecific band. DOX was administered either short- or long-tem as indicated. (TIF) [file pbio.1002330.s005.tif]

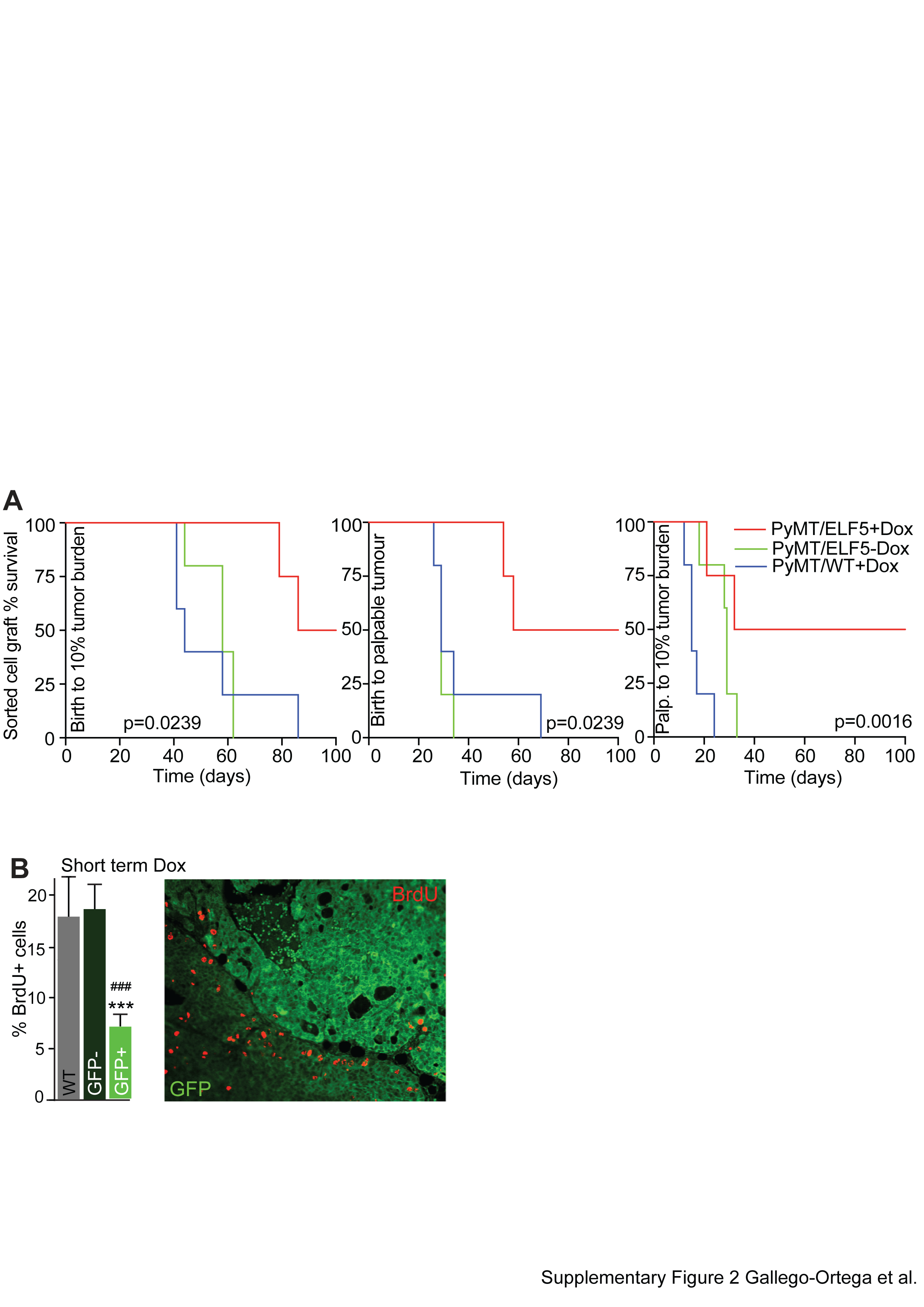

Supplement: S2 Fig — Panel A, survival analysis of animals carrying tumors that developed from intraductal transplantation of EGFP+ tumor cells made fluorescent by 7 d administration of DOX, then withdrawing DOX as indicated. The ELF5 transgenic cassette is not selective of a specific epithelial population during tumor progression showed by survival analysis. Panel B, proliferation after 7 d DOX treatment measured by BrdU incorporation (red cells) in EGFP high (bright green) compared to EGFP low/no areas (dark green) of primary tumors, quantified by counting cells in random fields (bar chart). (TIF) [file pbio.1002330.s006.tif]

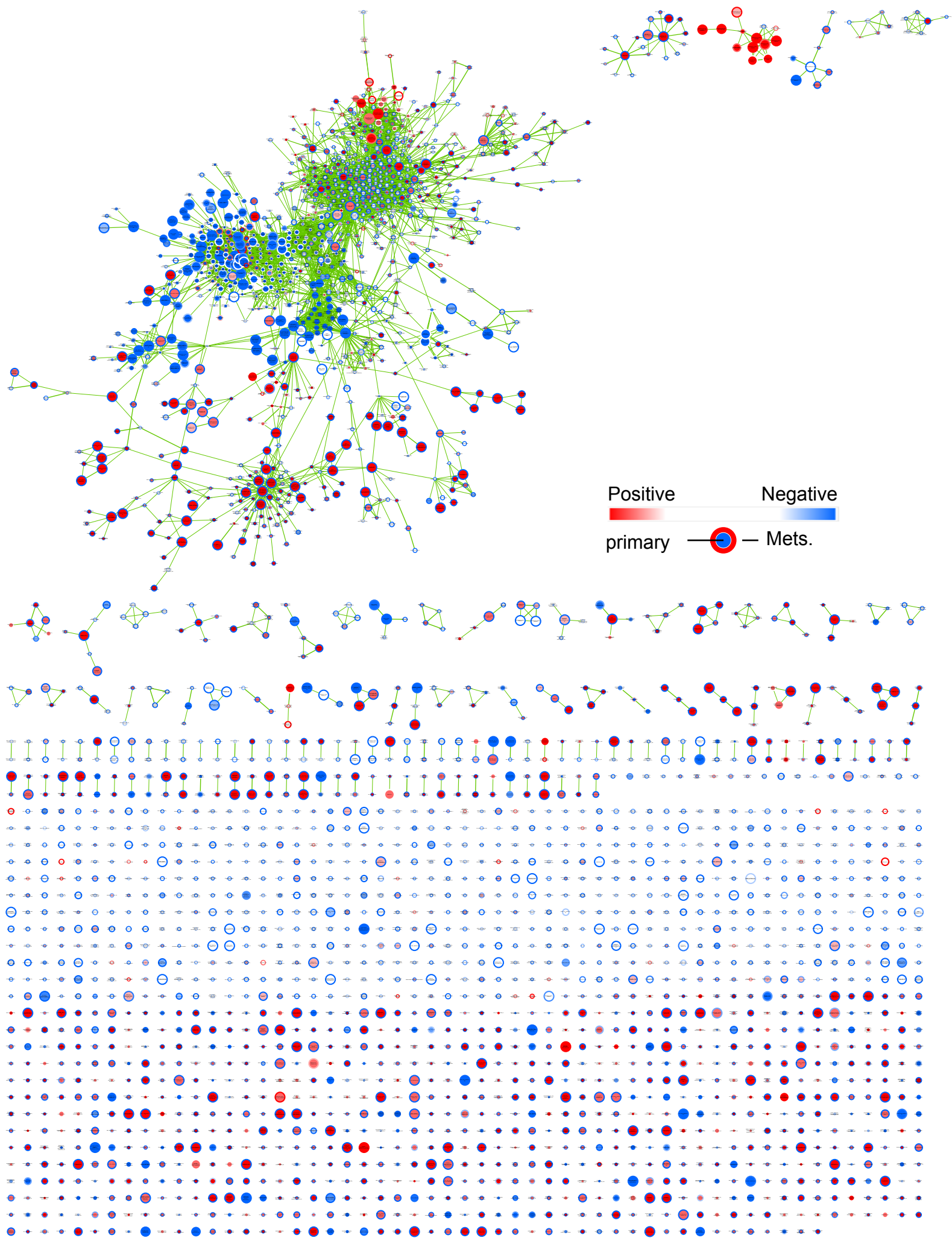

Supplementary Figure 3 Gallego-Ortega et al.

Supplement: S3 Fig — Figure can be viewed at a range of high magnifications, 1,600% or higher, to identify individual gene sets and to see the composition of functional clusters. (PDF) [file pbio.1002330.s007.pdf]

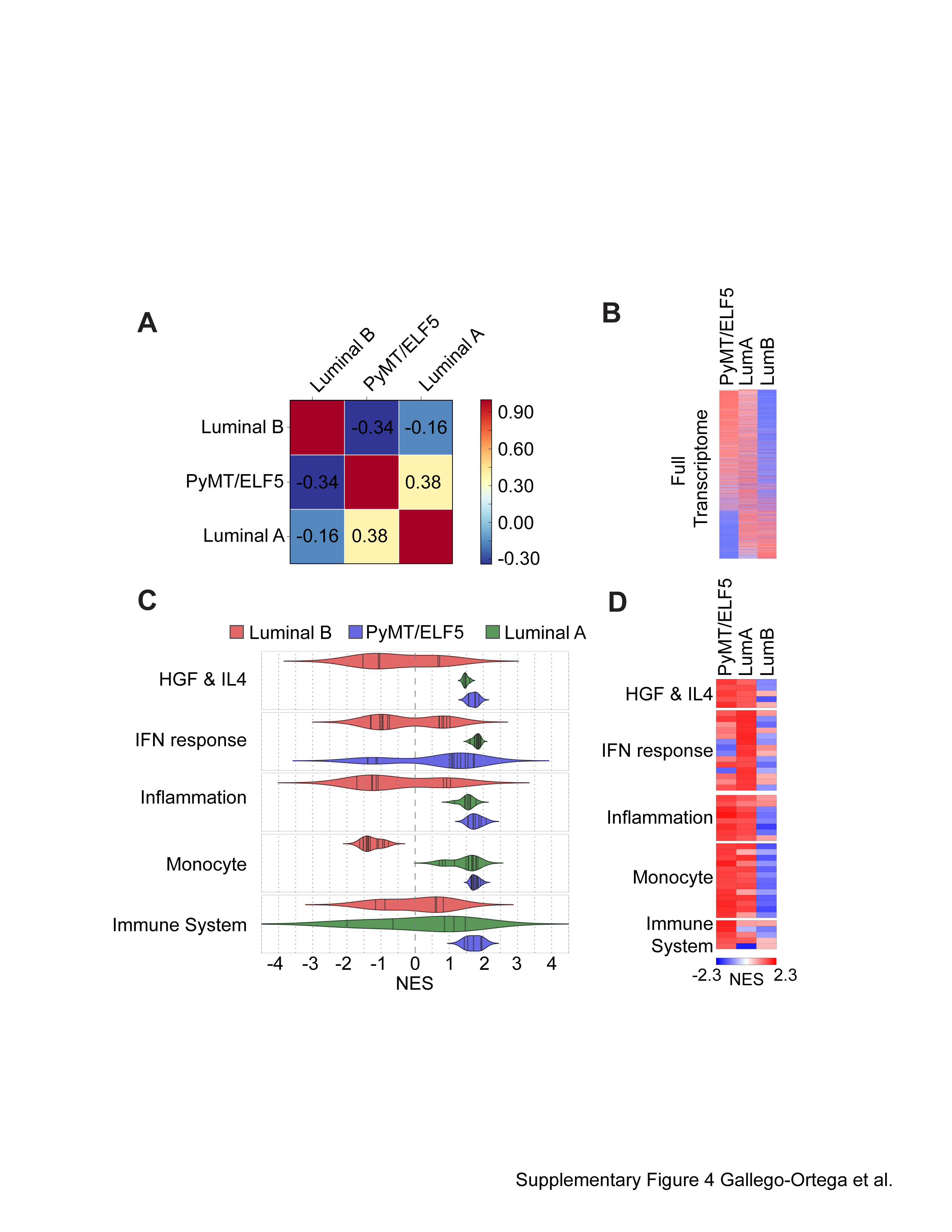

Supplement: S4 Fig — Differential gene expression associated with ELF5 expression in PAM50 defined Luminal A and B breast cancer was calculated and ranked (by LIMMA moderated t-statistic) and used as input for GSEA. Panel A, shows the Pearson correlation matrix between the normalized enrichment scores (NES) for all gene-sets. Panel B, heatmap of the full GSEA-derived transcriptome for Elf5 action in each luminal subtype of the TCGA series compared with the PyMT model, where each row represents the NES of a gene-set and are sorted by PyMT/ELF5 NES. Panel B, comparison of the defined inflammatory functional networks by GSEA enrichment scores in each luminal subtype of the TCGA series compared with the PyMT model. Panel C, heatmap showing the NES for each individual gene set included in the defined functional clusters. Gene-set names and statistics can be found in S1 Table. (TIF) [file pbio.1002330.s008.tif]

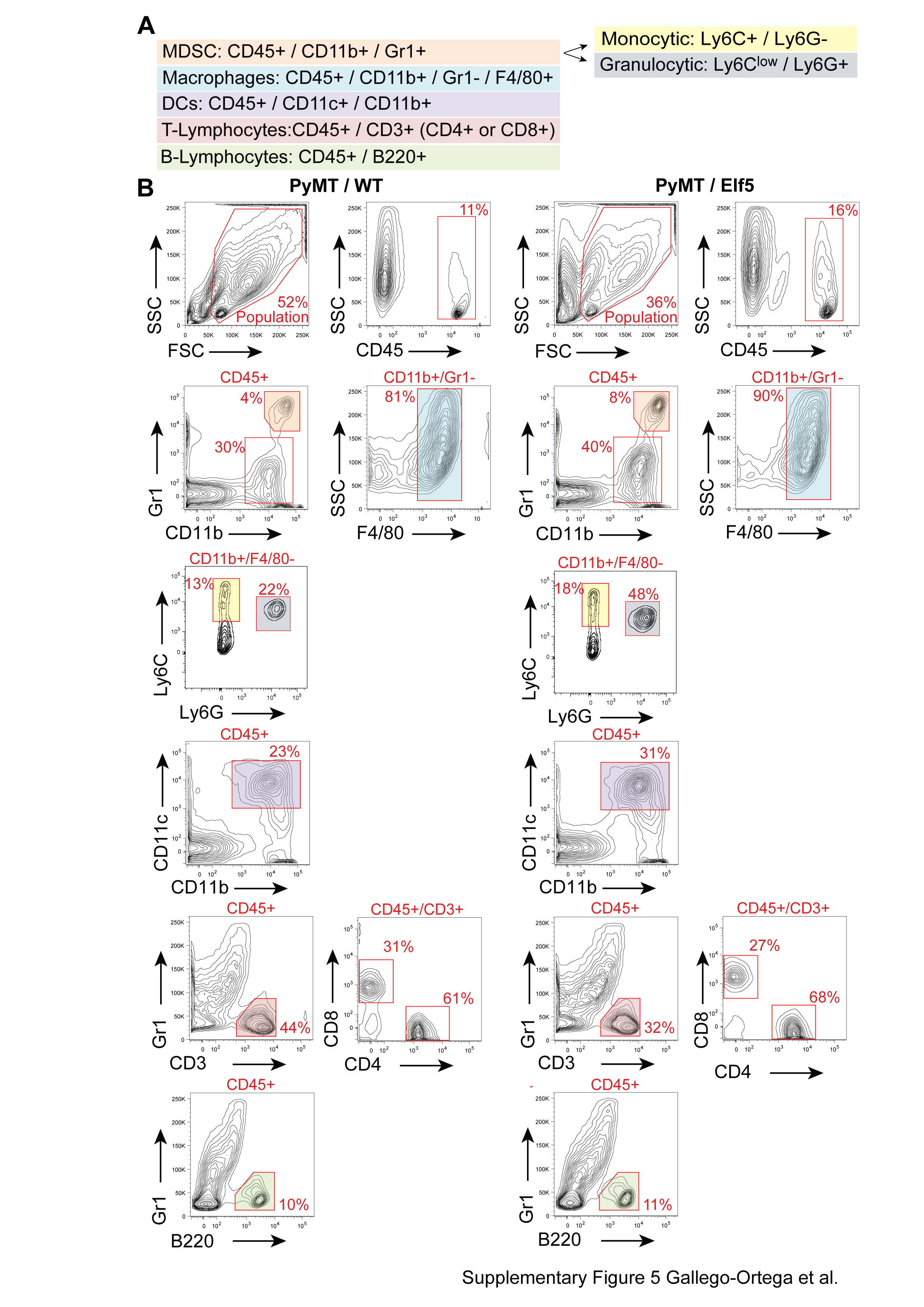

Supplement: S5 Fig — Panel A, definition of the cell sets used in this analysis. Panel B, gating strategy. Color coding of antibodies from panel A shows the gated populations they selected. (TIF) [file pbio.1002330.s009.tif]

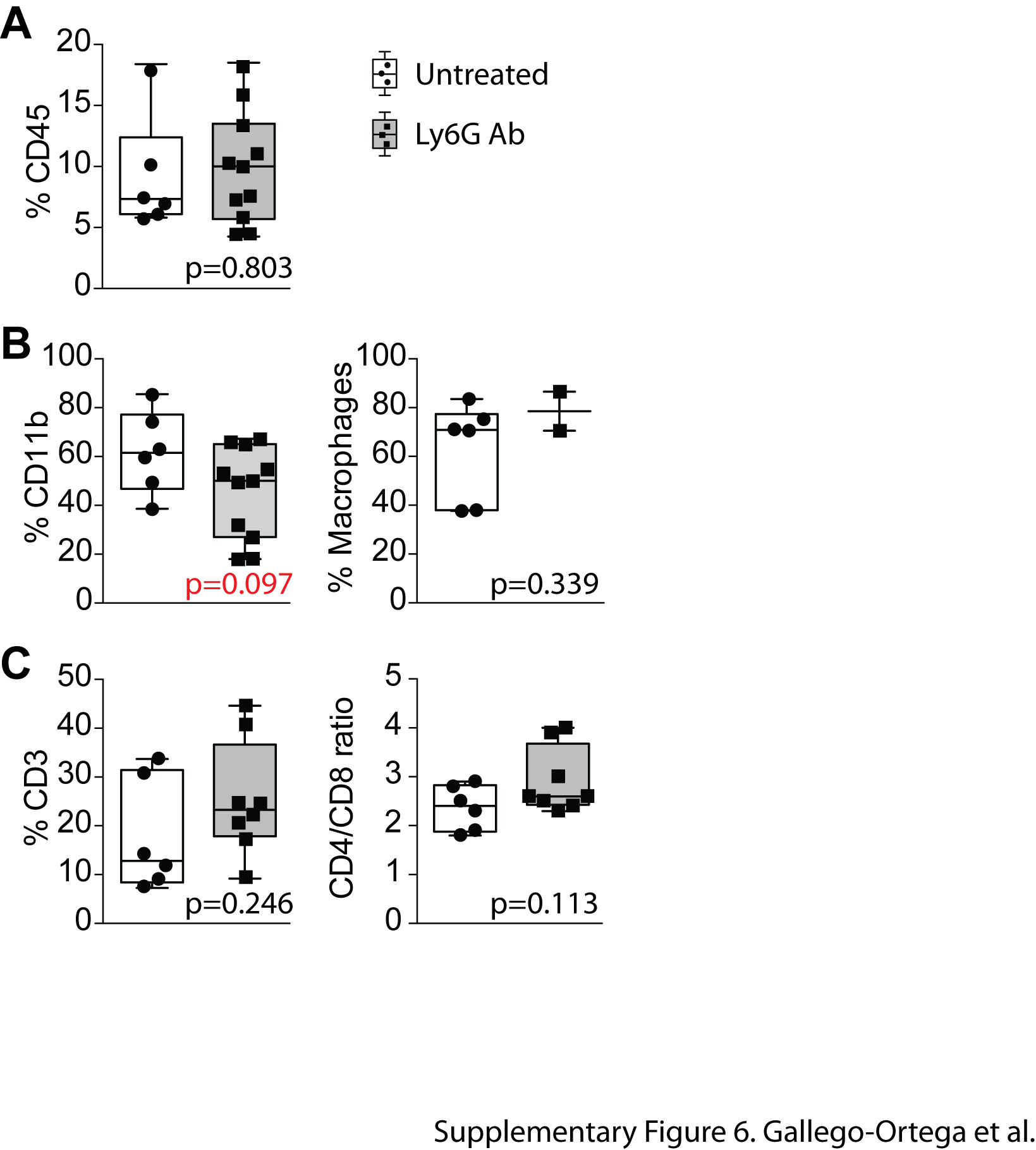

Supplement: S6 Fig — FACS analysis of immune infiltrates in tumors from PyMT/WT mice after Ly6G antibody treatment. Panel A, shows total leukocytes; Panel B, myeloid lineage; and Panel C T lymphocytes. (TIF) [file pbio.1002330.s010.tif]

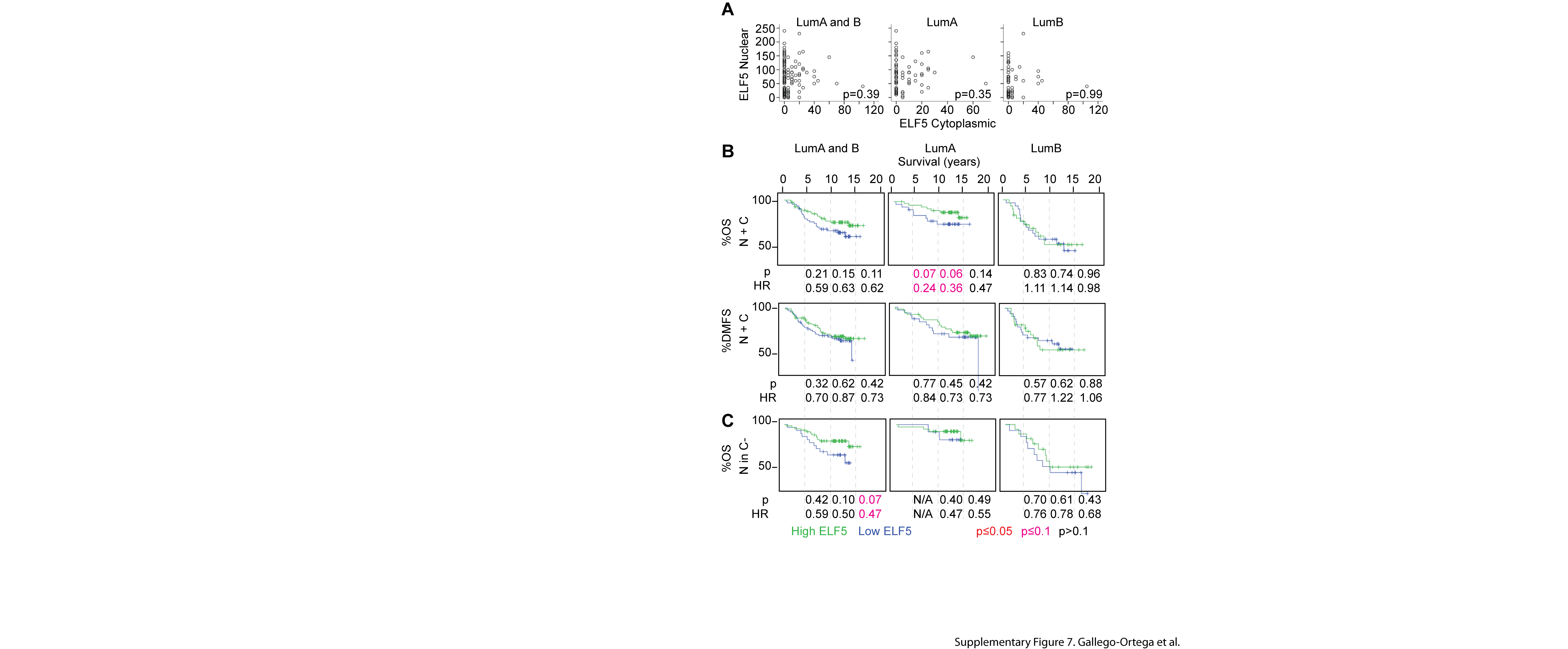

Supplement: S7 Fig — Panel A, correlation between cytoplasmic and nuclear staining in the analyzed patient cohort. Panel B, prognostic value (OS, overall survival and DMFS, distal metastasis free survival) of the combined cytoplasmic and nuclear ELF5 staining. Panel C, prognostic value in the samples positive for nuclear staining only. (TIF) [file pbio.1002330.s011.tif]
